# Minimum distance estimation of mean and standard deviation from reported quantiles

**DOI:** 10.1017/rsm.2026.10090

**Published:** 2026-04-15

**Authors:** Xiaoyu Tang, Tiejun Tong, Xin Zhang, Haitao Chu

**Affiliations:** 1 Academy of Pharmacy, Xi’an Jiaotong-Liverpool University, China; 2 Department of Mathematics, Hong Kong Baptist University, Hong Kong, China; 3 Data Sciences and Analytics, Pfizer Inc., China; 4 Division of Biostatistics and Health Data Science, University of Minnesota Twin Cities, USA

**Keywords:** five-number summary, meta-analysis, quantiles, sample mean, standard deviation

## Abstract

Meta-analysis is a cornerstone of evidence synthesis, yet challenges arise when studies report heterogeneous summary statistics, such as means and standard deviations (SDs) versus medians, interquartile ranges (IQRs), or other percentiles. Excluding studies that report only medians and IQRs can introduce bias and reduce precision, particularly when outcomes are skewed, which is common in clinical research. Although several methods exist to estimate means and SDs from alternative summaries, many rely on strong normality assumptions, exhibit computational burden, or fail to adequately account for the precision of reported quantiles (e.g., extreme values versus medians). To address these limitations, we propose two flexible weighted estimators for estimating the mean and SD from reported quantiles. The methods leverage inverse-variance and inverse–variance–covariance weighting, respectively, to enhance both accuracy and precision. Additionally, our methods are flexible enough to accommodate any set of reported quantiles and various underlying distributions, and they can be readily implemented using standard statistical software. Simulation studies demonstrate that the weighted estimators provide nearly unbiased estimates of the mean and SD with high precision in most cases, especially for large sample sizes. In a real-world meta-analysis, the estimates obtained using the proposed estimators closely aligned with those derived from true sample statistics. These approaches are particularly valuable for skewed outcomes and offer a practical and user-friendly solution for researchers seeking to integrate heterogeneous data while improving accuracy and precision.

## Highlights

### What is already known?

- Existing meta-analyses frequently encounter challenges when integrating studies that report different summary statistics (e.g., means and standard deviations [SDs] versus medians, interquartile ranges, or ranges). Such heterogeneity complicates the integration of quantile-based summaries with studies reporting means and SDs.
- Because the mean remains one of the primary metrics for evidence synthesis, there is an ongoing need for reliable methods to convert quantile-based summaries into means and SDs, even when outcomes are skewed.
- Although several estimation methods exist, many rely on normality assumptions, exhibit computational burden, or fail to adequately account for the differing precision of reported quantiles.

### What is new?

- We introduce weighted estimators for deriving means and SDs from reported quantiles, which significantly reduce bias and improve precision compared to existing approaches.
- Our framework is highly flexible, compatible with any set of quantiles, adaptable to various underlying distributions, and straightforward to implement in standard statistical software.

### Potential impact for *RSM* readers

- We provide a flexible and user-friendly solution to estimate means and SDs from quantiles for a wide range of distributions. By improving accuracy and precision, these methods enable the inclusion of studies reporting varying summaries in meta-analyses, thereby enhancing the robustness and reliability of research synthesis.

## 1 Introduction

Meta-analysis is an essential statistical tool for synthesizing evidence across studies. It enables researchers to draw more robust conclusions and enhances the generalizability of findings across various disciplines, including medicine, psychology, and environmental science.^1^ However, challenges arise when studies report different types of summary statistics for continuous outcomes. While some studies report means and standard deviations (SDs), others, especially those involving skewed data such as time-to-event outcomes,^2^ ^–^ ^4^ report medians with interquartile ranges (IQRs), ranges, or five-number summaries (minimum, first quartile, median, third quartile, and maximum).^5^ The inconsistency in reported summaries is a well-recognized challenge in literatures.

A commonly used but restrictive strategy is to exclude studies reporting medians and IQRs or ranges and to include only studies reporting means and SDs. However, this exclusion can significantly reduce the number of eligible studies, thereby increasing the risk of bias and diminishing precision in the pooled estimate, an issue that is especially problematic when the underlying distribution of sample data is skewed.^3^ ^,^ ^5^ These limitations have motivated the development of more refined methods to estimate means and SDs from alternative summary statistics.

One of the earliest and most widely cited methods was proposed by Hozo et al., who derived simple formulas based on the median, minimum, and maximum values.^6^ Brand later extended this approach to accommodate five-number summaries.^7^ Although pioneering, these methods rely heavily on extreme values and make only limited use of sample size information, resulting in reduced accuracy.^8^ Subsequent work by Wan et al., Luo et al., and Shi et al. derived improved analytical formulas for optimal estimators of sample means and SDs that fully use sample sizes and handle IQRs, ranges, and five-number summaries.^8^ ^–^ ^10^ However, their methods assume normally distributed data. To address this, Shi et al. also proposed methods for detecting skewness.^11^

When skewness is present, nonnormal conversion methods can improve accuracy. Kwon and Reis proposed a simulation-based estimator using approximate Bayesian computation to accommodate both normal and skewed distributions,^12^ ^,^ ^13^ but these approaches are computationally intensive and sensitive to the choice of tuning parameters. Shi et al. developed analytical formulas tailored to lognormal data, but these formulas are complex and not generalizable to other skewed distributions.^14^ McGrath et al. and Cai et al. introduced the methods based on the Box-Cox (BC) transformation,^15^ ^,^ ^16^ which offer greater flexibility but require the data to be transformable to approximate normality; otherwise, the resulting estimates may be biased. The BC-based methods also do not apply when summary statistics contain negative values.

Among existing methods, the quantile estimation (QE) approach proposed by McGrath et al. is particularly appealing due to its conceptual simplicity and ease of implementation.^15^ However, the QE method does not account for the varying precision of different quantiles, especially extreme values like the minimum and maximum, which often exhibit high variance. Ignoring this variability can limit both the accuracy and precision of the converted means and SDs.

The continued reliance on the means and SDs in meta-analysis underscores an ongoing need for robust conversion methods, even when the underlying data are skewed. This demand is reflected by the widespread use of existing conversion approaches. For instance, a Google Scholar search on November 11, 2025, showed that the paper presenting McGrath et al.’s QE method has already been cited 743 times, highlighting the practical importance of converting quantiles to means and SDs in applied research, even for skewed data. Therefore, advancing this area by developing more precise and efficient estimators is crucial for improving the quality and completeness of evidence synthesis.

In this article, we propose two weighted versions of the QE method based on the minimum distance estimation theory. These new estimators enhance finite-sample performance by preserving the core principles of the original QE approach while addressing its key limitations. The proposed methods are flexible across a wide range of underlying distributions, applicable to diverse set of quantile summaries, including those arising from growth curves, and straightforward to implement using standard statistical software. By leveraging the mathematical relationship between quantiles and the distributional parameters, our approach effectively converts reported quantiles into estimates of the mean and SD.

Specifically, we introduce weighted estimators that incorporate either the variances or the full variance–covariance matrix of the quantiles. Incorporating these weights is theoretically more efficient and leads to improvements in both accuracy and precision.

The remainder of paper is organized as follows. Section 2 reviews existing optimal estimators for normally distributed data, optimal estimators for lognormal data, and the QE method for skewed distributions. Section 3 describes our proposed weighted estimators. Section 4 presents simulation studies evaluating their performance relative to existing methods. Section 5 illustrates the application of our estimators using a real-world example, highlighting differences in pooled estimates obtained using different conversion techniques. Section 6 summarizes our findings and discusses the advantages, practical values, and limitations of the proposed estimators.

## 2 Existing methods

### 2.1 Normal data

Let $X_{1},X_{2},\ldots ,X_{n}$ be a random sample of size n from a normal distribution. The summary statistics commonly reported in the literature include $S_{1}=\{a,m,b;n\}$ , $S_{2}=\{q_{1},m,q_{3};n\}$ , and $S_{3}=\{a,q_{1},m,q_{3},b;n\}$ , where $a,q_{1},m,q_{3},b$ denote the minimum, first quartile, median, third quartile, and maximum, respectively. Luo et al. developed unbiased estimators that minimize the mean squared error (MSE) of sample mean from $S_{1}$ , $S_{2}$ , and $S_{3}$ .^9^ For scenario $S_{3}$ , the estimator of the sample mean is

(1) $$\begin{array}{r} \overset{―}{X}=\left(\frac{2.2}{2.2+n^{0.75}}\right)\frac{a+b}{2}+\left(0.7-\frac{0.72}{n^{0.55}}\right)\frac{q_{1}+q_{3}}{2}+\left(0.3+\frac{0.72}{n^{0.55}}+\frac{2.2}{2.2+n^{0.75}}\right)m. \end{array}$$

Wan et al. proposed nearly unbiased estimators to estimate the sample SD from $S_{1}$ and $S_{2}$ .^8^ Shi et al. later proposed an unbiased estimator with minimized MSE using $S_{3}$ as follows^10^:

(2) $$\begin{array}{r} S=\left(\frac{1}{1+0.07n^{0.6}}\right)\frac{\left(b-a\right)}{\xi }+\left(\frac{0.07n^{0.6}}{1+0.07n^{0.6}}\right)\frac{\left(q_{3}-q_{1}\right)}{\eta }, \end{array}$$

where $\xi =2\Phi^{-1}[(n-0.375)/(n+0.25)]$ , $\eta =2\Phi^{-1}[(0.75n-0.125)/(n+0.25)]$ , and $\Phi^{-1}$ is the quantile function of the standard normal distribution. The estimators above are widely used to transform quantiles in meta-analysis. However, these estimators assume that the data follow a normal distribution—an assumption that may not hold in many practical settings.

### 2.2 Skewed data

#### 2.2.1 Optimal transformation for lognormal data

Shi et al. proposed methods to estimate the mean and variance of a lognormal distribution using $S_{1}$ , $S_{2}$ , and $S_{3}$ .^14^ For scenario $S_{3}$ , the estimator of the sample mean is

(3) $$\begin{array}{r} \overset{―}{X}=\exp\left(\hat{\mu }+\frac{\sigma^{2}}{2}\right){\left(1+\frac{0.405}{n}{\hat{\sigma }}^{2}+\frac{0.315}{n}{\hat{\sigma }}^{4}\right)}^{-1}, \end{array}$$

where

(4) $$\begin{array}{rl} \hat{\mu } & =\left(\frac{2.2}{2.2+n^{0.75}}\right)\frac{\ln\left(a\right)+\ln(b)}{2}+\left(0.7-\frac{0.72}{n^{0.55}}\right)\frac{\ln\left(q_{1}\right)+\ln\left(q_{3}\right)}{2}\\ & \;+\left(0.3+\frac{0.72}{n^{0.55}}+\frac{2.2}{2.2+n^{0.75}}\right)\ln(m) \end{array}$$

and

(5) $$\begin{array}{r} {\hat{\sigma }}^{2}={\left[\left(\frac{1}{1+0.07n^{0.6}}\right)\frac{\ln\left(b\right)-\ln(a)}{\xi }+\left(\frac{0.07n^{0.6}}{1+0.07n^{0.6}}\right)\frac{\ln\left(q_{3}\right)-\ln\left(q_{1}\right)}{\eta }\right]}^{2}\times {\left(1+\frac{0.28}{{\left(\ln(n)\right)}^{2}}\right)}^{-1}. \end{array}$$

Accordingly, the estimator for the sample variance is

(6) $$\begin{array}{rl} {\tilde{\sigma }}^{2} & =\exp\left(2\hat{\mu }+2\hat{\sigma }\right){\left(1+\frac{1.62}{n}{\hat{\sigma }}^{2}+\frac{5.04}{n}{\hat{\sigma }}^{4}\right)}^{-1}\\ & \;-\exp\left(2\hat{\mu }+{\hat{\sigma }}^{2}\right){\left(1+\frac{1.62}{n}\hat{\sigma }+\frac{1.26}{n}{\hat{\sigma }}^{4}\right)}^{-1}. \end{array}$$

The derivations and calculations for these estimators are complicated, and the method is limited to the lognormal distribution.

#### 2.2.2 The QE method

McGrath et al. proposed the QE method, which allows estimations from various underlying distributions for scenarios $S_{1}$ , $S_{2}$ , and $S_{3}.$ ^15^

The QE method selects several candidate parametric distributions, including the normal, lognormal, gamma, beta, and Weibull distributions. For scenario $S_{3}$ , the parameters of each distribution are estimated by minimizing:

(7) $$\begin{array}{r} S\left(\theta \right)={\left(F_{\theta }^{-1}\left(\frac{1}{n}\right)-a\right)}^{2}+{\left(F_{\theta }^{-1}(0.25)-q_{1}\right)}^{2}+{\left(F_{\theta }^{-1}(0.5)-m\right)}^{2}+{\left(F_{\theta }^{-1}(0.75)-q_{3}\right)}^{2}+{\left(F_{\theta }^{-1}\left(1-\frac{1}{n}\right)-b\right)}^{2}, \end{array}$$

where $F_{\theta }^{-1}$ denotes the quantile function of the distribution parameterized by θ . The distribution yielding the smallest value of $S(\hat{\theta })$ is taken as the underlying distribution. The sample mean and SD are then estimated from the parameters of the selected distribution.

## 3 Proposed weighted estimators

The QE method does not account for the variance of quantiles and treats all quantiles as equally reliable, which can limit its efficiency and precision. To address this limitation, we propose two flexible and user-friendly weighted estimators. These methods accommodate a wide range of parametric distributions and adapt to any available quantile information, thereby enhancing the robustness and efficiency for estimating means and SDs.

Let $X_{i,n}$ be the $100P_{i}$ -th empirical percentile obtained from a sample of size n where $0<P_{i}<1$ for i=1,2,…,k ( k≥3 ), where k is the number of available quantiles. k=3 if the summary statistics $S_{1}$ or $S_{2}$ are obtained, and k=5 if a five-number summary ( $S_{3}$ ) is observed. We assume that the data follow a parametric distribution characterized by the cumulative distribution function (CDF) F(X;β) and probability density function (PDF) f(X;β) with the parameters $\beta =(\beta_{1},\beta_{2},\ldots ,\beta_{J})$ , with J≤k . We denote $X_{i}=F^{-1}(P_{i};\beta )$ , the theoretical quantile of the underlying distribution.

In general, our proposed method constructs weighted estimators to obtain an estimate of β (denoted by $\hat{\beta }$ ) from available quantile information. Then, the mean and SD can be directly estimated using $\hat{\beta }$ .

### 3.1 Weighted QE

We first introduce the weighted QE (wQE) method by incorporating weights for the quantiles. The objective function is defined as the sum of weighted squared differences between the observed quantiles $X_{i,n}$ and the corresponding theoretical quantiles estimated from the assumed underlying distribution. The estimated distributional parameters $\hat{\beta }$ are then obtained by minimizing the weighted objective function:

(8) $$\begin{array}{r} \sum_{i=1}^{k}w_{i}{\left(X_{i,n}-F^{-1}\left(P_{i};\beta \right)\right)}^{2}, \end{array}$$

where $w_{i}$ is the inverse of asymptotic variance of $\sqrt{n}(X_{i,n}-F^{-1}(P_{i};\beta ))$ , which is $f{\left(X_{i};\beta \right)}^{2}/[P_{i}(1-P_{i})]$ , where $f(X_{i};\beta )$ can be estimated by $f(F^{-1}(P_{i};\hat{\beta });\hat{\beta })$ .

### 3.2 Minimum distance estimator

We further extend the wQE method to a Minimum Distance Estimator (MDE) by incorporating the asymptotically optimal weighting scheme.^17^ Specifically, this approach accounts not only for the variances of the quantiles but also for their covariances. The parameters of the underlying distribution $\hat{\beta }$ are obtained by minimizing the corresponding weighted objective function:

(9) $$\begin{array}{r} \left(X_{1,n}-F^{-1}\left(P_{1};\beta \right),\ldots ,X_{k,n}-F^{-1}\left(P_{k};\beta \right)\right)W_{x}{\left(X_{1,n}-F^{-1}\left(P_{1};\beta \right),\ldots ,X_{k,n}-F^{-1}\left(P_{k};\beta \right)\right)}^{⊤}, \end{array}$$

where $W_{x}=\Omega_{x}^{-1}$ , and $\Omega_{x}$ is the asymptotic covariance matrix for $\sqrt{n}(X_{1,n}-F^{-1}(P_{1};\beta ),\ldots ,X_{k,n}-F^{-1}(P_{k};\beta ){)}^{⊤}$ . The (i,j) -th element of $\Omega_{x}$ is

(10) $$\begin{array}{r} n\mathrm{Cov}(X_{i,n},X_{j,n})=\frac{P_{i}(1-P_{j})}{f(X_{i};\beta )f(X_{j};\beta )}, \end{array}$$

where $f(X_{i};\beta )$ can be estimated by $f(F^{-1}(P_{i};\hat{\beta });\hat{\beta })$ .

### 3.3 Asymptotic behavior of weights for minimum and maximum

The weights for extremes (minimum and maximum) change with the sample size. Table 1 and Supplementary Text S1 summarize the asymptotic behavior of the weights for the minimum and maximum under the normal distribution with a mean μ and a SD σ , the lognormal distribution with a location parameter μ and a scale parameter σ , the gamma distribution with a shape parameter α and a rate parameter β , the beta distribution with shape parameters α and β , and the Weibull distribution with a shape parameter k and a scale parameter λ as the sample size increases. The PDFs of these distributions are in Supplementary Text S1.

**Table 1:** Asymptotic behavior of weights for minimum and maximum Table 1 Long description.

Distribution	Weight for minimum	Weight for maximum
Normal	O(1/n)	O(1/n)
Lognormal	$O(n^{-1+2\sigma \sqrt{2/\ln(n)}})$	$O(n^{-1-2\sigma \sqrt{2/\ln(n)}})$
Weibull	$O(n^{(2-k)/k})$	$O(\frac{{\left(\ln(n)\right)}^{2(k-1)/k}}{n})$
Gamma	$O(n^{(2-\alpha )/\alpha })$	O(1/n)
Beta	$O(n^{(2-\alpha )/\alpha })$	$O(n^{(2-\beta )/\beta })$

*Note*: Table 1 describes the asymptotic behavior of weights for minimum and maximum under the normal distribution with a mean μ and a SD σ , the lognormal distribution with a location parameter μ and a scale parameter σ , the gamma distribution with a shape parameter α and a rate parameter β , the beta distribution with shape parameters α and β , and the Weibull distribution with a shape parameter k and a scale parameter λ as the sample size increases. The probability density functions of these distributions are in Supplementary Text S1.

Theoretically, as n goes to infinity, the following patterns emerge. For the normal and lognormal distributions, the weights for both the minimum and maximum converge to zero. For the Weibull and gamma distributions, the weight for the maximum converges to zero. The behavior for the minimum, however, depends on the shape parameter: it converges to zero when the shape parameter exceeds 2, remains constant when equal to 2, and diverges to infinity when less than 2. A similar pattern holds for the beta distribution, where the asymptotic weight for the minimum depends on the shape parameter α and for the maximum on the shape parameter β . In general, the weight assigned to an extreme quantile reflects its informational contribution. For distributions with light tails or low density near the boundaries, extreme values (the minimum or maximum) tend to become highly variable in large samples, contributing more noise than signal about the central characteristics of the distribution; accordingly, their weights decrease. In contrast, for distributions with heavy tails or high boundary density, extreme observations remain informative for estimating distributional parameters, and their weights decrease more slowly or even increase. This weighting scheme demonstrates how our estimators adapt automatically to the changing reliability of quantiles as the sample size grows and under different distributional assumptions.

The theoretical possibility of infinite asymptotic weights does not impede implementation. In practice, with finite samples, all estimated weights are finite. Moreover, the weighted estimators depend only on the relative weights among quantiles. We may normalize the weights so that they sum to one without affecting the estimates, as is commonly done in matching adjusting indirect treatment comparisons and other survey sampling weighting approaches.^18^ ^,^ ^19^

### 3.4 Estimation procedure

To minimize the objective function, we solve for the point at which the gradient equals zero. However, due to the nonlinearity of the gradient equations and the absence of closed-form solutions, iterative optimization techniques are required. A commonly used approach is the quasi-Newton algorithm, which is well suited for nonlinear least squares problems. Additionally, the limited-memory BFGS algorithm for bound-constrained optimization (L-BFGS-B) approach can be employed to impose constraints on the parameter space.^20^ This method iteratively refines the parameter estimates until convergence criteria are satisfied. Once the parameters of the underlying distribution are estimated, the corresponding mean and variance can be computed directly.

Suppose we observe a five-number summary from data assumed to follow a beta distribution, as is common for health-related quality of life (HRQL) scores.^21^ In this context, F(X,β) denotes the CDF of the beta function. The PDF, CDF, and quantile function for the beta distribution can be easily calculated in R using dbeta(), pbeta(), and qbeta(), respectively. Using an iterative optimization procedure, we can estimate the shape parameters $\hat{\alpha }$ and $\hat{\gamma }$ . The sample mean can then be calculated as $\hat{\mu }=\hat{\alpha }/(\hat{\alpha }+\hat{\gamma })$ , and the sample variance can be calculated as ${\hat{\sigma }}^{2}=\hat{\alpha }\hat{\gamma }/[{\left(\hat{\alpha }+\hat{\gamma }\right)}^{2}(\hat{\alpha }+\hat{\gamma }+1)]$ . These formulas follow directly from the known properties of the beta distribution. Similar functionality exists in R for many other commonly used distributions, making the method broadly applicable. Additionally, software such as SAS, which also supports evaluation of density functions, CDFs, and quantile functions for a wide range of distributions, can be used to implement our approaches. This flexibility highlights the adaptability of our method to different distributional assumptions supported by standard statistical software. The R code to implement our models is available at https://github.com/xiaoyu9411/MDE.

In practice, the underlying data distribution is often unknown. Following the approach of the QE method, we can select from a set of candidate parametric distributions, such as the normal, lognormal, gamma, beta, and Weibull, by identifying the one that minimizes the sum of squared residuals between the reported and theoretical quantiles. As an alternative, minimizing the sum of absolute residuals can also be employed for this distribution selection.

### 3.5 Percentiles for the minimum and maximum

The percentile information for minimum and maximum that the QE method used is 1/n and 1−(1/n) , respectively. However, the true percentile of the minimum lies within [0, 1/n ], and the percentile of the maximum lies within [1−(1/n),1] .^22^ For normal distributions, theoretical percentiles can be approximated using Blom’s equation, which assigns 0.625/(n+0.25) for the minimum and (n−0.375)/(n+0.25) for the maximum.^23^ For simplicity, we approximate Blom’s equation using 0.625/n for the minimum and 1−(0.625/n) for the maximum. In our simulation studies (Section 4), we examined the performance of the QE method under three percentile specifications for the minimum: 1/n , 0.5/n, and 0.625/n (correspondingly with 1−(1/n) , 1−(0.5/n) , and 1−(0.625/n) for the maximum), where 0.5/n is the midpoint of range [0, 1/n ]. We adopted the percentile with the best performance for our proposed estimators.

## 4 Simulation studies

### 4.1 Data generation and scenarios

**Figure 1:**
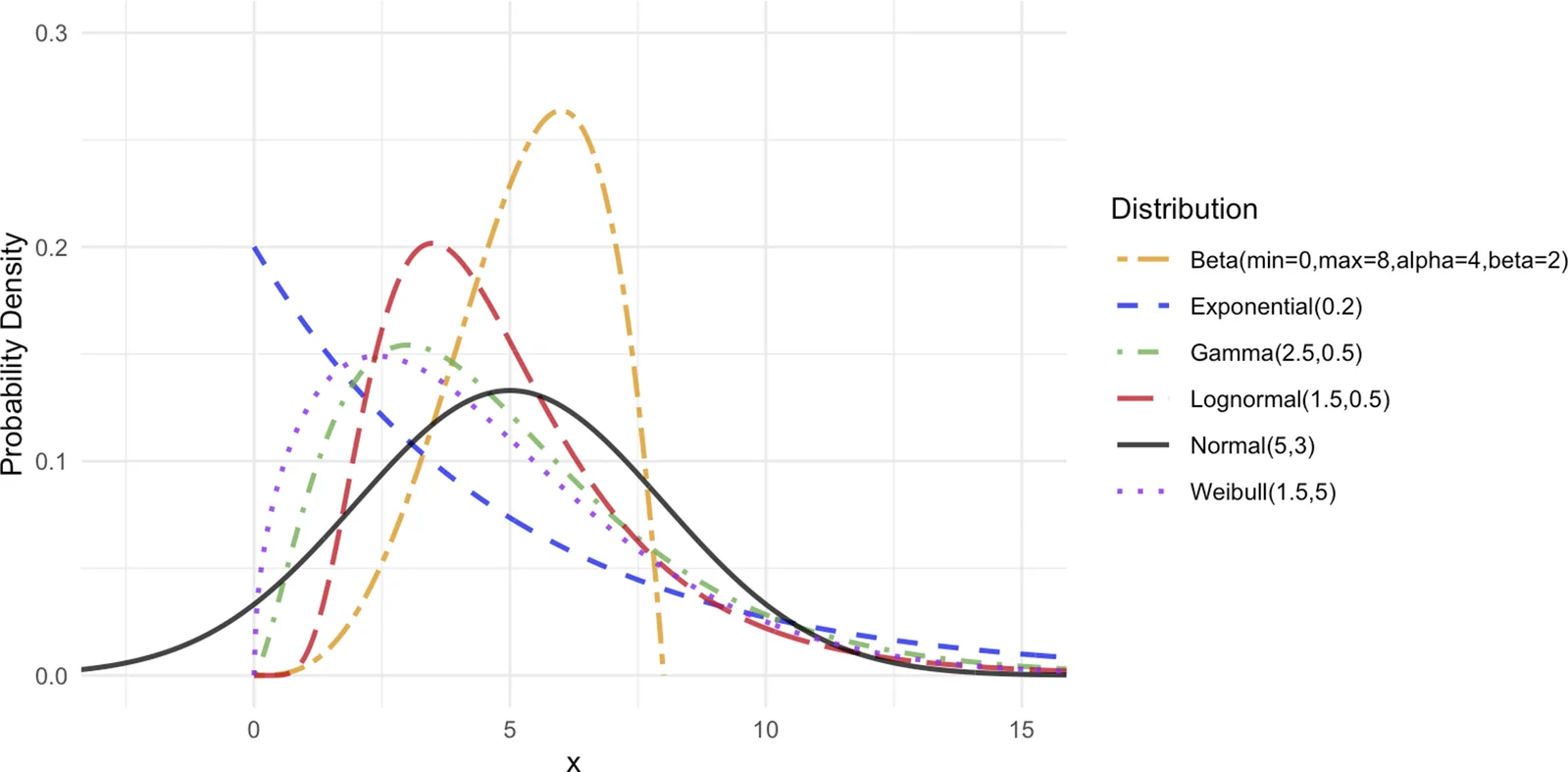
Distributions in simulation studies. Figure 1 Long description.

We considered six different underlying distributions in our simulations (Figure 1): (1) a scaled beta distribution within the range [0,8] with shape parameters α=4 and β=2 ; (2) a Weibull distribution with a shape parameter k=1.5 and a scale parameter λ=5 ; (3) a normal distribution with a mean μ=5 and a SD σ=3 ; (4) a lognormal distribution with a location parameter μ=1.5 and a scale parameter σ=0.5 ; (5) a gamma distribution with a shape parameter α=2.5 and a rate parameter β=0.5 ; and (6) an exponential distribution with a rate parameter λ=0.2 . The parameter configurations were selected to ensure that the resulting means and SDs are broadly comparable across distributions while capturing a range of distributional shapes, including left-skewed, right-skewed, and symmetric forms. The corresponding values of the means and SDs are presented in Supplementary Table S1.

Sample sizes ranged from 30 to 1,000 in increments of 10. We also considered both least squares and least absolute deviations for selecting the underlying distributions in our proposed methods across the five candidate distributions mentioned above. For each combination of distribution, sample sizes, and distribution-selection method, we conducted 10,000 iterations under scenarios $S_{1}$ , $S_{2},$ and $S_{3}$ .

To assess the performance of our proposed models under the conditions where theoretical asymptotic weights may diverge (as described in Section 3.3), we conducted additional simulations under $S_{3}$ with least squares method for model selection. Specifically, we examined (1) a Weibull distribution with a shape parameter k=0.8 and a scale parameter λ=5 ; (2) a gamma distribution with a shape parameter α=0.8 and a rate parameter β=5 ; (3) a beta distribution with shape parameters α=0.8 and β=4 ; and (4) a beta distribution with shape parameters α=4 and β=0.8 .

To evaluate the performance of different methods, we calculated the relative bias of the estimated mean ( $\hat{\mu }$ ) and the estimated SD ( $\hat{\sigma }$ ) with respect to their true values, defined as follows:

(11) $$\begin{array}{r} \mathrm{RB}\left(\hat{\mu }\right)=\frac{1}{T}\sum_{r=1}^{T}\frac{{\hat{\mu }}_{r}-\mu }{\mu },\mathrm{RB}\left(\hat{\sigma }\right)=\frac{1}{T}\sum_{r=1}^{T}\frac{{\hat{\sigma }}_{r}-\sigma }{\sigma }, \end{array}$$

and the relative mean squared error (RMSE) as follows:

(12) $$\begin{array}{r} \mathrm{RMSE}\left(\hat{\mu }\right)=\frac{\frac{1}{T}\sum_{r=1}^{T}{\left({\hat{\mu }}_{r}-\mu \right)}^{2}}{\frac{1}{T}\sum_{r=1}^{T}{\left({\overset{―}{X}}_{r}-\mu \right)}^{2}},\mathrm{RMSE}\left(\hat{\sigma }\right)=\frac{\frac{1}{T}\sum_{r=1}^{T}{\left({\hat{\sigma }}_{r}-\sigma \right)}^{2}}{\frac{1}{T}\sum_{r=1}^{T}{\left(S_{r}-\sigma \right)}^{2}}, \end{array}$$

where T is the number of iterations, $\overset{―}{X}$ stands for the true sample mean, and S represents the true sample SD.

### 4.2 Results

#### 4.2.1 Choice of percentiles for the minimum and maximum

We examined the performance of the QE method under three percentile specifications for the minimum: 1/n , 0.5/n , and 0.625/n (correspondingly with 1−(1/n) , 1−(0.5/n) , and 1−(0.625/n) for the maximum) under $S_{3}$ (Supplementary Figures S1–S4). When the underlying distributions are scaled, beta(4,2) , normal(5,3) , lognormal(1.5,0.5) , and gamma(2.5,0.5) using 0.625/n and 0.5/n as percentiles for minimum significantly reduced both bias and RMSEs for estimates of the mean and SD compared to 1/n . Under Weibull(1.5,5) , both 0.625/n and 0.5/n slightly improved relative bias for the mean and SD and RMSEs in SD for large sample sizes (Supplementary Figure S5). Under exponential(0.2) , both 0.625/n and 0.5/n reduced relative bias for the mean and SD and RMSEs in SD for small sample sizes (Supplementary Figure S6). Given the small differences in performance between 0.5/n and 0.625/n , we prioritized theoretical alignment with Blom’s method, adopting 0.625/n as the percentile for the minimum and 1−(0.625/n) as the percentile for the maximum.

#### 4.2.2 Comparison of different methods

**Figure 2:**
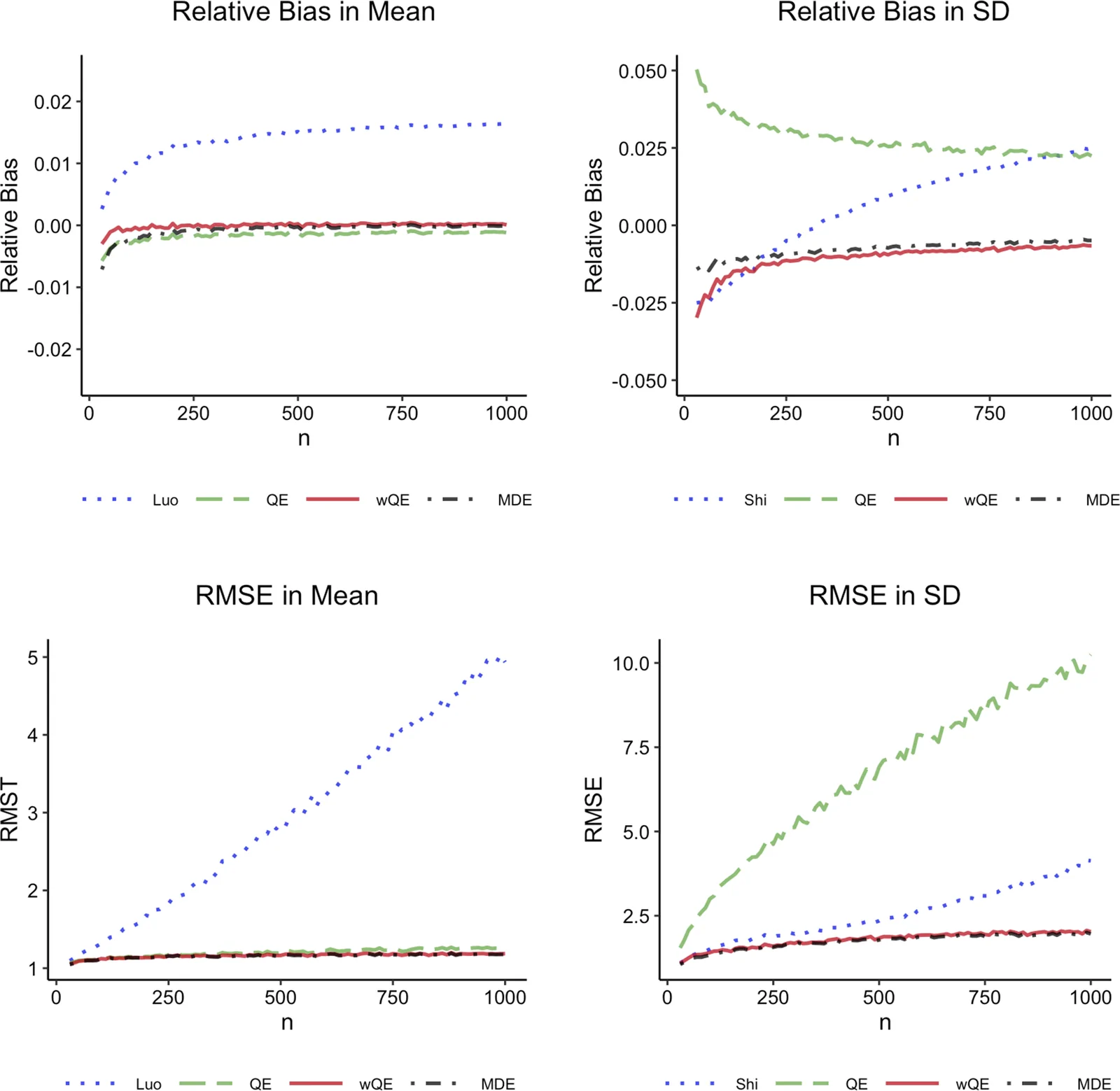
Performance of estimators under Beta(min=0,max=8,α=4,β=2) in $S_{3}$ . Least squares method was used for distribution selection for wQE and MDE. Figure 2 Long description.

**Figure 3:**
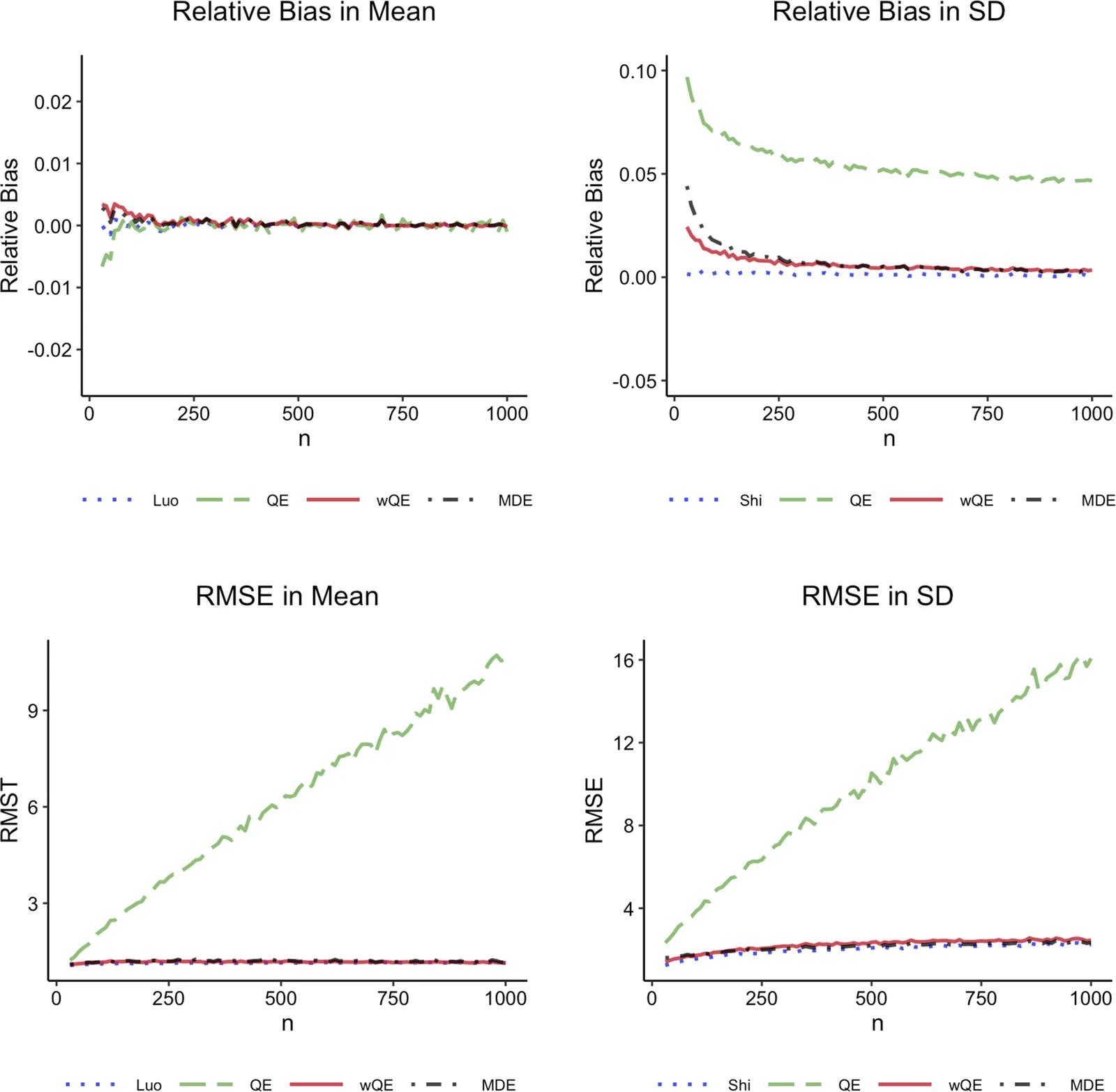
Performance of estimators under Normal(5,3) in $S_{3}$ . Least squares method was used for distribution selection for wQE and MDE. Figure 3 Long description.

**Figure 4:**
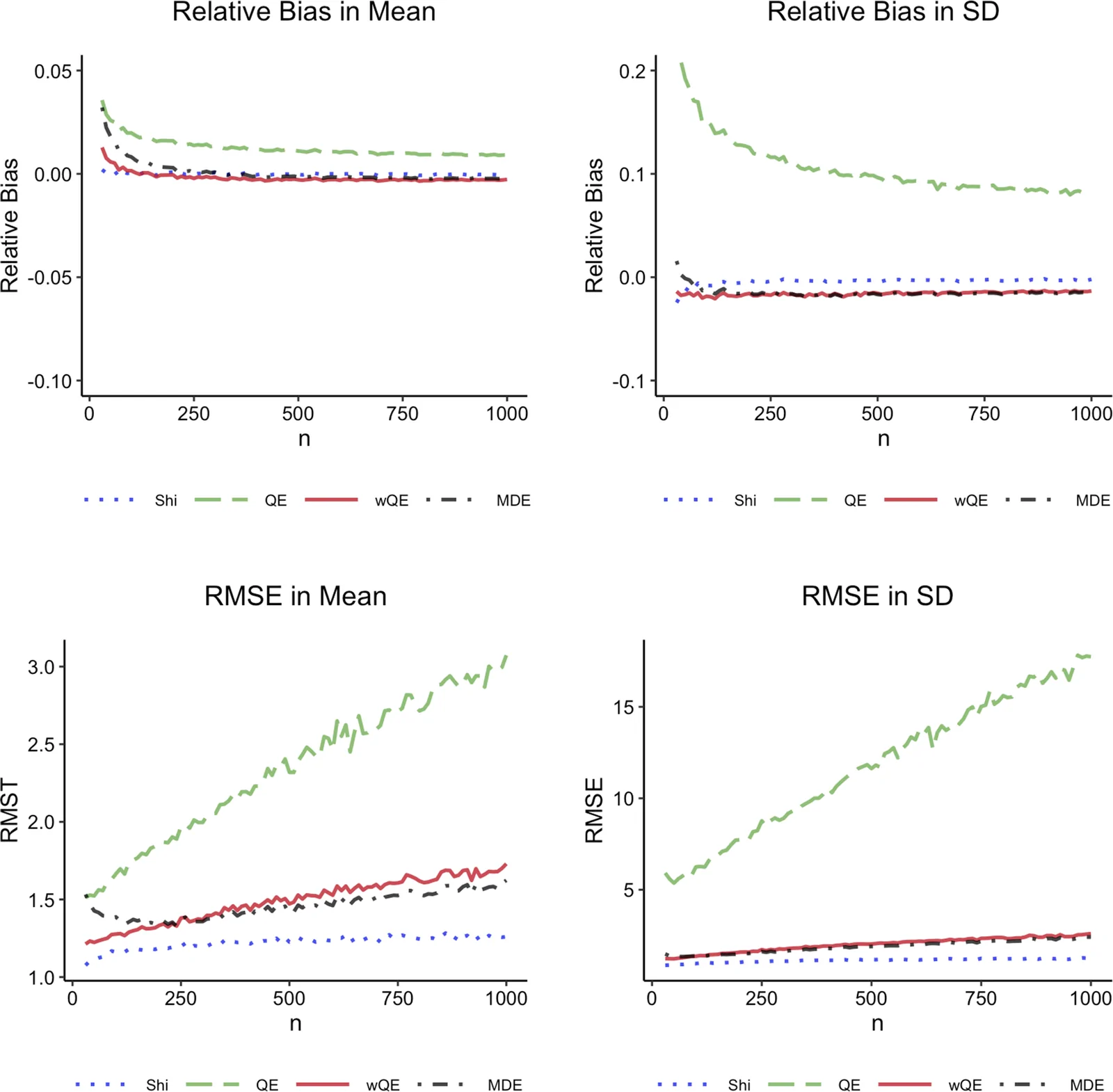
Performance of estimators under Lognormal(1.5,0.5) in $S_{3}$ . Least squares method was used for distribution selection for wQE and MDE. Figure 4 Long description.

**Figure 5:**
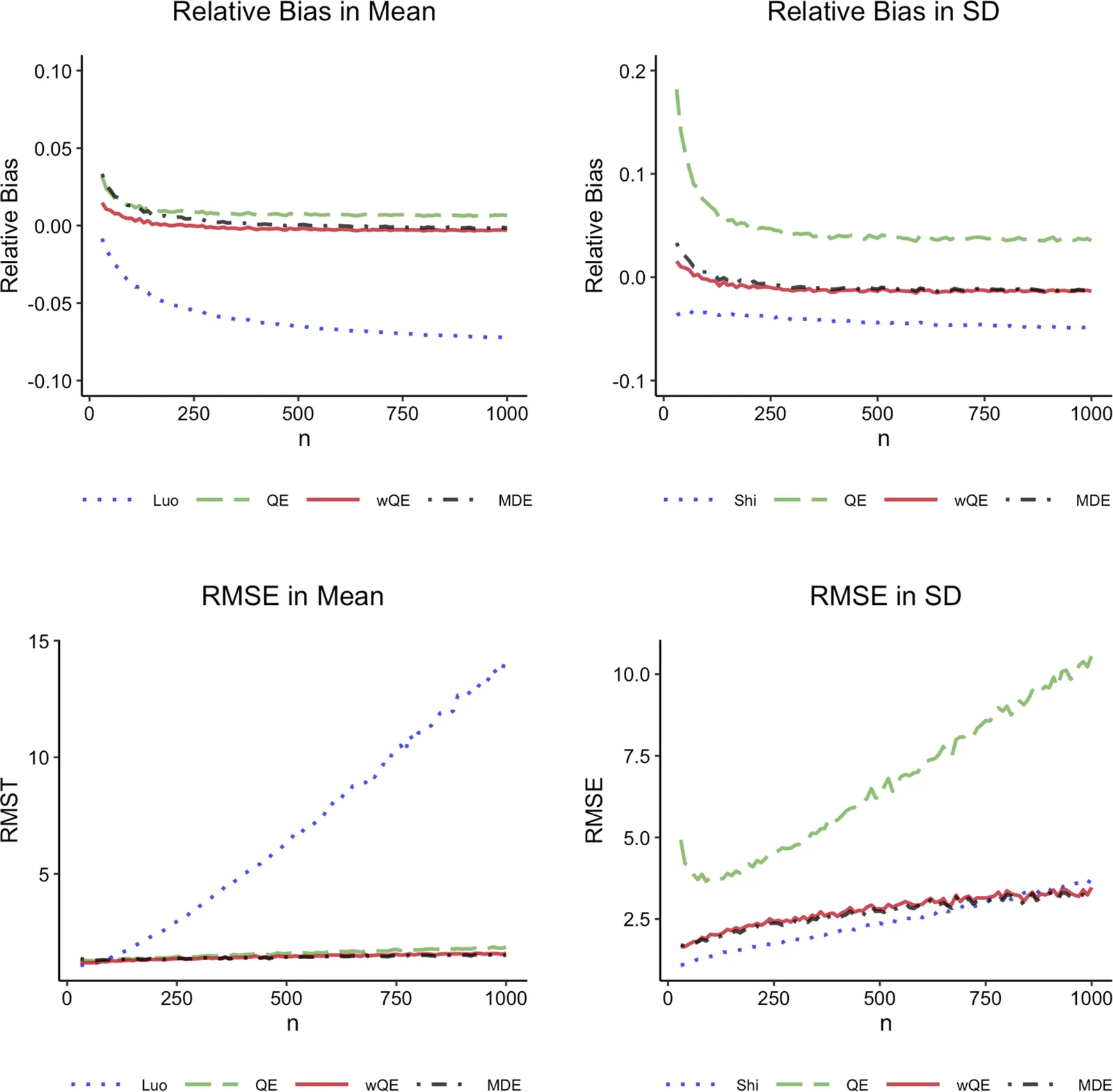
Performance of estimators under Gamma(2.5,0.5) in $S_{3}$ . Least squares method was used for distribution selection for wQE and MDE. Figure 5 Long description.

**Figure 6:**
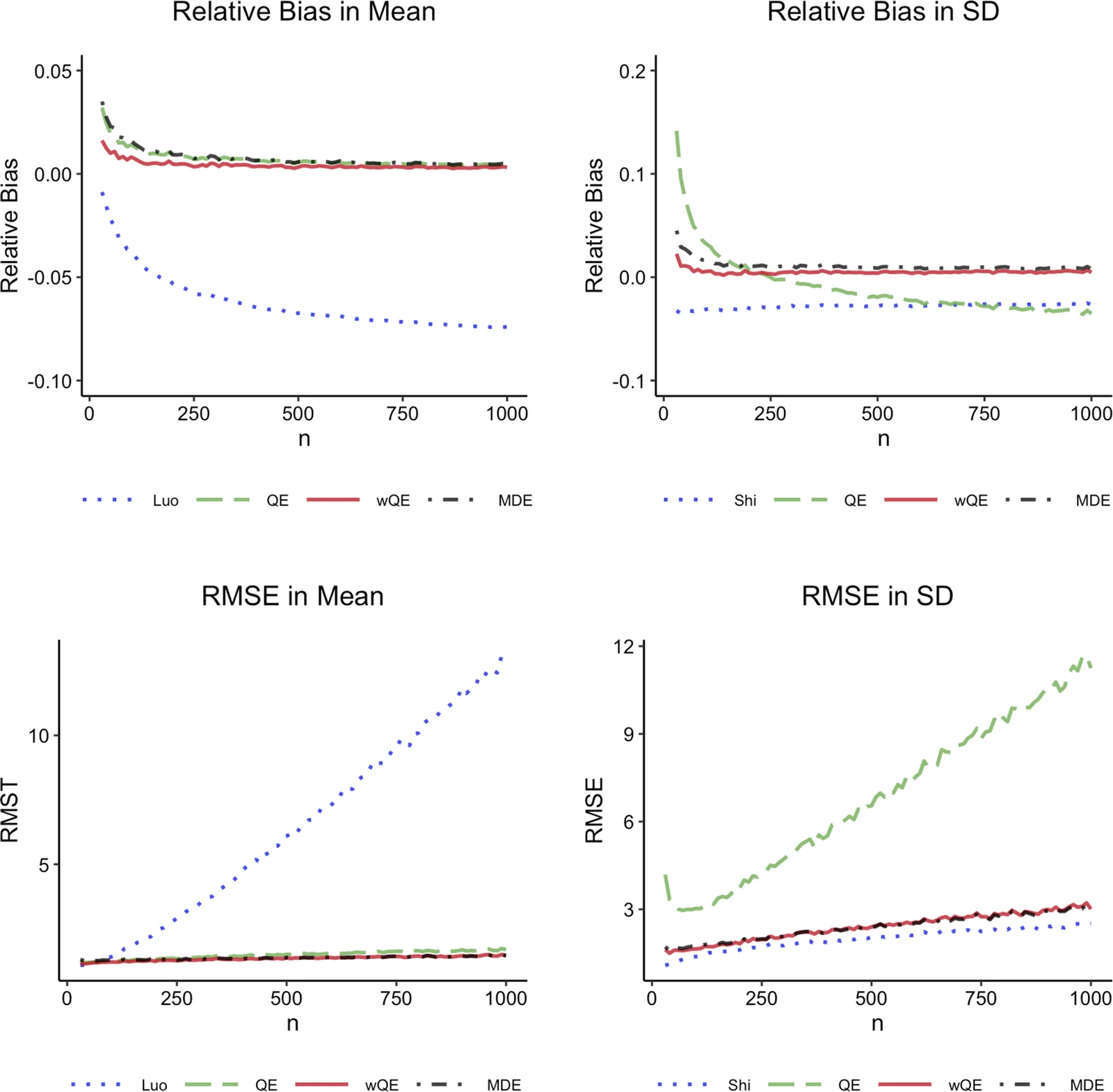
Performance of estimators under Weibull(1.5,5) in $S_{3}$ . Least squares method was used for distribution selection for wQE and MDE. Figure 6 Long description.

**Figure 7:**
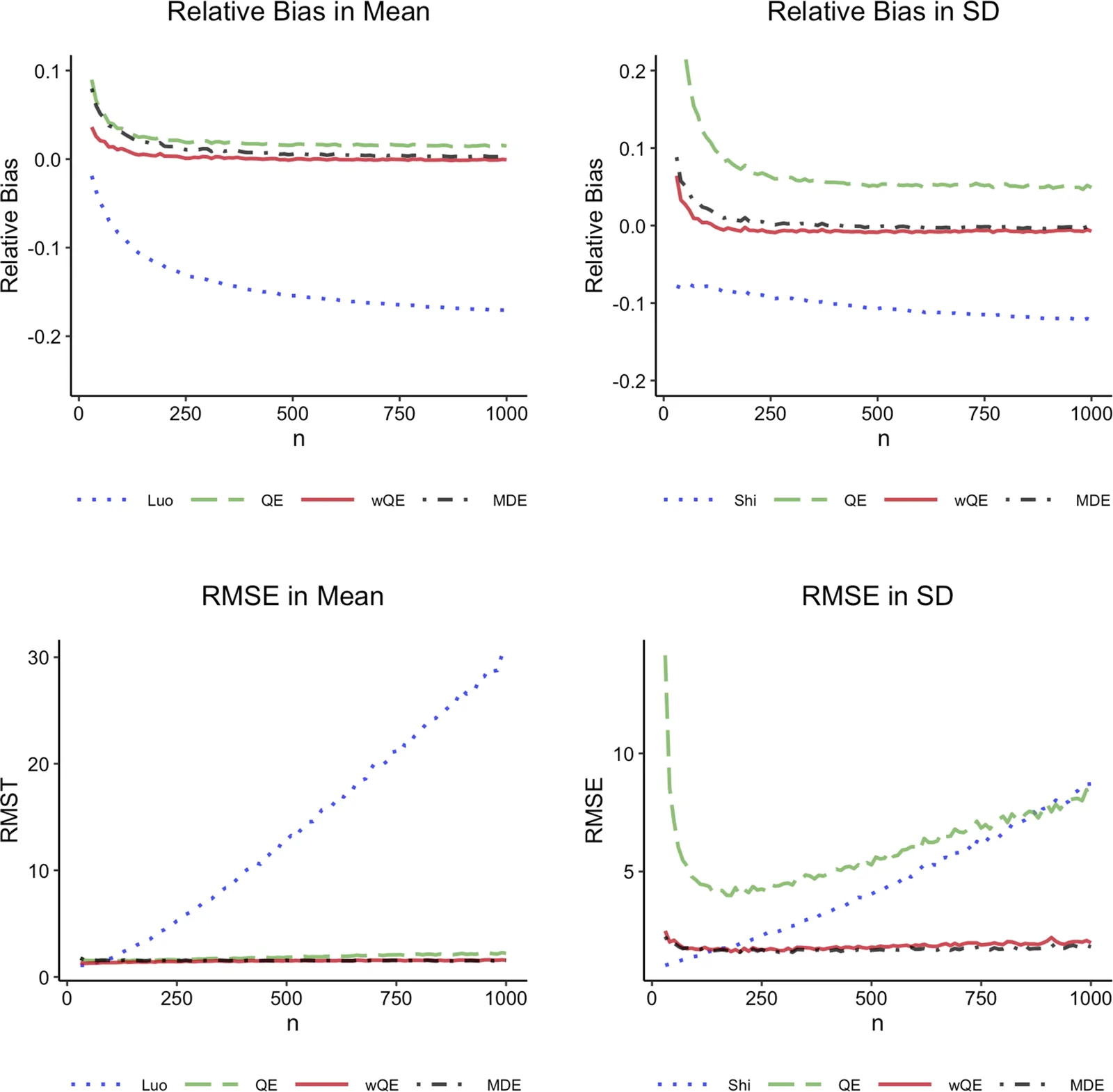
Performance of estimators under Exponential (0.2) in $S_{3}$ . Least squares method was used for distribution selection for wQE and MDE. Figure 7 Long description.

Results under scenario $S_{3}$ are presented in Figures 2–7. Both the wQE and MDE methods outperformed the QE method across all six underlying distributions, with a particular advantage for SD estimates where a dramatic reduction in RMSEs was observed. The two proposed methods exhibited similar performance in general, but the MDE method showed slightly higher relative bias compared to the wQE method in some cases. The method by Luo et al. and Shi et al. yielded biased estimates for the scaled beta(4,2) , gamma(2.5,0.5) , Weibull(1.5,5) , and exponential(0.2) distributions. For the normal(5,3) distribution, our methods provided mean estimates comparable to Luo’s optimal method for the mean, but exhibited slightly higher bias for the SD when sample sizes were smaller than 100 compared to Shi’s optimal method for the SD. The RMSEs for both the mean and SD are comparable to the optimal methods. Under the lognormal(1.5,0.5) distribution, our methods slightly overestimated the mean and SD for sample sizes below 200 and showed higher RMSE compared to Shi’s method, which was optimal for this distribution. For the remaining distributions, both wQE and MDE outperformed the optimal methods based on the normal distribution. When sample size was less than 100, the bias for our methods remained within 10% for exponential(0.2) and within 4% for other distributions; for larger sample sizes, our methods generated nearly unbiased estimates with low RMSE.

The results in $S_{1}$ were consistent with the findings from scenario $S_{3}$ (Supplementary Figures S7–S12). wQE and MDE also outperformed the QE method. The pattern of performance against the optimal methods was similar. In $S_{1}$ , our methods exhibited the highest precision for the scaled beta(4,2) , gamma(2.5,0.5) , Weibull(1.5,5) , and exponential(0.2) distributions.

Results under scenario $S_{2}$ are presented in Supplementary Figures S13–S18. The optimal methods for normal distribution yielded biased estimates for the scaled beta(4,2) , gamma(2.5,0.5) , Weibull(1.5,5) , and exponential(0.2) distributions. The wQE and MDE methods performed similarly to the QE method overall. When the sample size was small, all three methods overestimated the mean and SD for the scaled beta(4,2) , normal(5,3) , gamma(2.5,0.5) , Weibull(1.5,5) , and exponential(0.2) distributions and underestimated the mean and SD for the lognormal(1.5,0.5) distribution. For large sample sizes, the estimates were nearly unbiased in most of distributions, with the exception of slight underestimation of the SD for the lognormal(1.5,0.5) distribution and a slight overestimation of the SD for the Weibull(1.5,5) and exponential(0.2) distributions. For the normal(5,3) distribution, the RMSE of the mean for our methods was higher than that of QE for *n* < 600 but became lower as the sample size increased. For the exponential(0.2) distribution with n<200 , our methods yielded substantially lower relative bias and RMSE for the SD than the QE method.

In distribution selection, the proportion of correct selection increases with sample size in general (Supplementary Figures S19–S21). Our methods achieved higher correct selection rates for the normal distribution in $S_{2}$ (60%–100%) compared to the QE method (50%–90%). For the Weibull(1.5,5) distribution, our methods achieved correct selection rates of 50%–80% in $S_{1}$ and 40%–70% in $S_{3}$ , whereas the QE method rarely selected the correct distribution (under 20% in $S_{1}$ and $S_{3}$ ). For the exponential distribution, our methods achieved higher correct selection rates than the QE method in both $S_{1}$ and $S_{3}$ when the sample size was below 400. The QE method demonstrated a higher proportion of correct selections for the lognormal and gamma distributions in $S_{1}$ and $S_{3}$ . wQE, MDE, and QE showed comparable overall performance in other scenarios. We also evaluated the use of least absolute deviations for distribution selection but found it did not improve performances and reduced the correct selection rate for the normal distribution (Supplementary Figures S22–S42); therefore, the least squares criterion was adopted for our models.

Under distributions where the theoretical asymptotic weights may diverge (Supplementary Figures S43–S46), our methods generally achieved lower relative bias and RMSE for both the mean and SD than the QE approach across nearly all scenarios. For large sample sizes, the proposed estimators produced nearly unbiased estimates across the examined distributions. Overall, the two proposed methods performed similarly, although the wQE method demonstrated slightly lower relative bias and RMSE than the MDE method in certain cases.

## 5 Illustrative example

We illustrated our proposed methods using the data from an individual participant data meta-analysis that investigated the diagnostic accuracy of Patient Health Questionaire-9 (PHQ-9), which was used as an illustrative example in the QE paper by McGrath et al.^15^ ^,^ ^24^ For each study, they provided the sample mean, sample SD, and five-number summaries (Supplementary Table S2). The PHQ-9 is a self-administered questionnaire that assesses the severity of depressive symptoms, with scores ranging from 0 to 27. Higher scores indicate more severe depression. Prior research has found that PHQ-9 scores tend to be right-skewed.^25^ ^,^ ^26^ Although the original meta-analysis focused on diagnostic accuracy using bivariate modeling, the availability of IPD allowed for the calculation of the true sample means and SDs for each study. This provided an opportunity to benchmark the performance of conversion methods against actual values, serving as a validation exercise within a real-world research context. Additionally, the IPD enabled us to generate all five-number summaries, illustrating our method’s application across different scenarios under $S_{1}$ , $S_{2}$ , and $S_{3}$ . Using our proposed methods and other existing methods mentioned above, we meta-analyzed PHQ-9 scores from 58 primary studies. Given the bounded and right-skewed distribution of PHQ-9 scores, we normalized the five-number summaries by dividing each value by 27. This transformation constrained the values within a range of 0–1, making them suitable for modeling using the beta distribution. After estimating the parameter estimation of the chosen distribution, we rescaled the derived sample means and SDs by multiplying them by 27, thereby reverting to the original scale of PHQ-9 scores.

We used a random-effects model for meta-analysis with between-study variance estimated using restricted maximum likelihood.^27^ Table 2 presents the meta-analysis of PHQ-9 scores, using both true sample means/SDs and estimates derived from quantiles across different methods under $S_{1}$ , $S_{2}$ , and $S_{3}$ . The benchmark results, obtained from the *true sample means and SDs*, yielded a pooled PHQ-9 estimate of 6.53 (95% CI: 5.97, 7.09), with a between-study variance (τ) of 2.13 and $I^{2}$ of 97.4%. Under $S_{1}$ , the QE method produced a pooled estimate of 7.65 (95% CI: 7.08, 8.23), representing the largest deviation from the benchmark. In contrast, the wQE and MDE methods yielded estimates of 6.50 (95% CI: 5.93, 7.07) and 6.51 (95% CI: 5.93, 7.09), which were closer to the value obtained from the true sample means and SDs. All methods yielded slightly higher heterogeneity estimates compared to the benchmark ( τ : 2.15–2.33; $I^{2}$ : 97.5%–98.5%). The QE method exhibited a predominant selection of the beta distribution (93.1%), while the wQE and MDE methods demonstrated a balanced selection between Weibull distribution and beta distribution (Supplementary Table S3). In $S_{2}$ , the Luo/Shi’s method showed a considerable underestimation (5.68; 95% CI: 5.06, 6.29). The QE, wQE, and MDE methods all showed better performance, with MDE (6.60; 95% CI: 5.90, 7.30) being closest to the benchmark. These three methods exhibited higher estimates of $I^{2}$ (97.9%–98.1%) and τ (2.58–2.68) compared to the benchmark. The distribution selection patterns in $S_{2}$ were diverse across all methods, with no single distribution dominating (Supplementary Table S4). Under $S_{3}$ , the Luo/Shi’s method demonstrated the poorest performance (5.97; 95% CI: 5.36, 6.58). The QE method showed the closest results to the benchmark (6.59; 95% CI: 6.03, 7.15), with wQE and MDE producing slightly higher estimates (6.65; 95% CI: 6.11, 7.20 and 6.71; 95% CI: 6.16, 7.25, respectively). Heterogeneity statistics for these methods were consistent with those derived from the true sample means and standard deviations ( τ : 2.07–2.13; $I^{2}$ : 97.1%–97.9%). wQE and MDE showed strong preference for beta distribution (82.8% and 84.5%), while the QE method showed comparable selection rates for Weibull (43.1%) and beta (41.4%) distributions (Supplementary Table S5). Given the stable performance of wQE and MDE methods across all scenarios, they are valuable for skewed data conversion.

**Table 2:** Meta-analysis results on PHQ-9 scores based on different conversion methods Table 2 Long description.

Scenario	$S_{1}$			$S_{2}$			$S_{3}$		
	Pooled			Pooled			Pooled		
	estimate			estimate			estimate		
Methods	(95% CI)	τ	$I^{2}$	(95% CI)	τ	$I^{2}$	(95% CI)	τ	$I^{2}$
True sample	6.53	2.13	97.4%	6.53	2.13	97.4%	6.53	2.13	97.4%
mean/SD	(5.97, 7.09)			(5.97, 7.09)			(5.97, 7.09)		
Luo/Shi	5.76	2.33	98.5%	5.68	2.35	97.8%	5.97	2.32	98.1%
(Wan)	(5.15, 6.37)			(5.06, 6.29)			(5.36, 6.58)		
QE	7.65	2.15	97.5%	6.67	2.58	98.0%	6.59	2.13	97.5%
	(7.08, 8.23)			(5.99, 7.35)			(6.03, 7.15)		
wQE	6.50	2.18	97.9%	6.66	2.60	97.9%	6.65	2.07	97.1%
	(5.93, 7.07)			(5.97, 7.34)			(6.11, 7.20)		
MDE	6.51	2.21	98.0%	6.60	2.68	98.1%	6.71	2.08	97.8%
	(5.93, 7.09)			(5.90, 7.30)			(6.16, 7.25)		

*Note*: Luo/Shi (Wan) are the optimal methods based on normal distributions. QE refers to the quantile estimation method. wQE is the weighted QE method. MDE is the minimum distance estimator.

## 6 Discussion

In meta-analysis, researchers frequently need to convert quantile-based summaries into means and standard deviations to incorporate studies that reported different types of summary statistics. In this paper, we highlight the limitations of existing approaches and propose weighted estimators for estimating sample means and SDs from quantiles commonly reported in literatures. These estimators facilitate meta-analyses that integrate studies reporting quantiles with those reporting means and SDs. In addition, our methods are readily applicable to a wide range of underlying distribution for which PDFs and CDFs can be computed using standard software.

Closed-form formulas for optimal estimators of sample mean and SD exist for normal^8^ ^–^ ^10^ and lognormal distributions.^14^ In our simulations, while the proposed estimators exhibited slightly larger biases and RMSEs than these optimal methods for small sample sizes, their performance was comparable for larger sample sizes under most cases in $S_{1}$ and $S_{3}$ . For other skewed distributions examined, our methods outperformed the normal-based optimal methods in both relative bias and RMSE. The QE method assumes equal weighting across quantiles, but quantiles vary in reliability due to their varying variances. For example, the median is a robust statistic with a bounded influence function,^28^ whereas the minimum and maximum are more variable due to their sensitivity to extreme values. This motives the use of inverse-variance weighting in our proposed estimators. Consistent with this intuition, our simulations show that the weighted approaches provide improved accuracy over the QE method, particularly when the minimum and maximum are included. The two proposed methods performed similarly across almost all scenarios. Given their comparable performance, we recommend the wQE estimator for routine use due to its more intuitive theoretical foundation, particularly for clinicians and applied researchers.

Although meta-analyses often focus on group differences, for which the sampling distribution of the mean difference typically approaches normality via the Central Limit Theorem, even when the underlying data are nonnormal or skewed, the summary statistics themselves are typically reported separately by group, particularly for secondary or exploratory endpoints.^29^ The underlying data within each group may be substantially skewed. Furthermore, there is often a need to meta-analyze the means of raw outcome values themselves, not just differences, for example, when establishing reference ranges.^30^ Existing methods, especially those relying on analytical formulas, are generally limited to commonly reported summaries (e.g., $S_{1}$ , $S_{2},$ and $S_{3}$ ). However, with the growing adoption of distributed learning,^31^ it is increasingly feasible to obtain more detailed quantile data from participating sites while still preserving data privacy. In such settings, our proposed approaches are versatile and can accommodate a variety of underlying distributions and any set of quantiles, including commonly reported summaries or more detailed percentile data. We also recommend that future studies report additional percentiles beyond the minimum and maximum (e.g., the 5th and 95th percentiles), as extreme values may reflect outliers and are often less informative.

Our work has several limitations. First, we require the number of parameters ( J ) in the underlying distribution be less than or equal to the number of available quantiles ( k ). When J>k , the number of parameters exceeds the number of available quantiles, making them not estimable. However, since k is usually at least 3 in practical applications, our methods are broadly applicable to most parametric distributions, which generally require fewer than three parameters. Second, our simulation studies focus on sample sizes larger than 30, which reflect typical study sizes in meta-analyses. However, in certain contexts, such as research on rare diseases, meta-analyses may include studies with very small sample sizes (e.g., *n* ≈ 10). Because our methods rely on iterative optimization rather than closed-form equations, the performance can become unstable under such conditions. Third, we evaluated both least squares and least absolute deviations for distribution selection, but the correct selection rate remained below 50% for some distributions at small sample sizes likely due to the limited number of quantiles available. Hence, developing improved model selection criteria is an important direction for future work. We recommend conducting sensitivity analyses to assess how alternative distributional assumptions influence the meta-analysis results.

In conclusion, our methods offer a flexible framework for estimating sample mean and SD from quantiles across a variety of underlying distributions, facilitating more comprehensive meta-analysis that incorporate different summary formats. Researchers may either specify a plausible distribution based on subject-matter knowledge or use data-driven model selection to guide estimation.

## Supporting information

10.1017/rsm.2026.10090.sm001 Tang et al. supplementary material Tang et al. supplementary material

## Data Availability

The data supporting the illustrative example are included in the Supplementary Material. The R code implementing the proposed methods can be accessed at https://url.uk.m.mimecastprotect.com/s/Go1SCL866TPY5oX9FBfqSyHdaa?domain=zenodo.org.

## Supplementary material

To view supplementary material for this article, please visit http://doi.org/10.1017/rsm.2026.10090.

## Long descriptions

### Table 1 Long description

The table consists of three columns and five rows of data.

* Normal distribution: Weight for minimum is O (1 all over n). Weight for maximum is O (1 all over n).

* Lognormal distribution: Weight for minimum is O (n to the power of minus 1 plus 2 sigma square root of 2 all over ln (n)). Weight for maximum is O (n to the power of minus 1 minus 2 sigma square root of 2 all over ln (n)).

* Weibull distribution: Weight for minimum is O (n to the power of (2 minus k) all over k). Weight for maximum is O (numerator (ln (n)) to the power of 2 (k minus 1) all over k, all over denominator n).

* Gamma distribution: Weight for minimum is O (n to the power of (2 minus alpha) all over alpha). Weight for maximum is O (1 all over n).

* Beta distribution: Weight for minimum is O (n to the power of (2 minus alpha) all over alpha). Weight for maximum is O (n to the power of (2 minus beta) all over beta).

Navigate back to Table 1.

### Figure 1 Long description

The x-axis represents a variable x ranging from 0 to 15 and the y-axis represents Probability Density from 0.0 to 0.3.

Six distinct curves are plotted:

* Beta(min=0, max=8, alpha=4, beta=2): A yellow dashed line with the highest peak of approximately 0.26 at x=6, dropping sharply to zero at x=8.

* Exponential(0.2): A blue dashed line starting at its highest point of 0.2 at x=0 and decaying asymptotically toward the x-axis.

* Gamma(2.5, 0.5): A green dash-dot line peaking at approximately 0.15 near x=3.

* Lognormal(1.5, 0.5): A red long-dashed line peaking at approximately 0.2 near x=4.

* Normal(5, 3): A solid black line forming a wide bell shape peaking at approximately 0.13 at x=5.

* Weibull(1.5, 5): A purple dotted line peaking at approximately 0.14 near x=2.5.

All curves except the Exponential and Normal distributions start at the origin (0,0). The legend is positioned on the right side of the plot area.

Navigate back to Figure 1.

### Figure 2 Long description

A four-panel line graph set. All panels share an x-axis labeled n, ranging from 0 to 1000.

Top-left panel: Relative Bias in Mean. The y-axis ranges from negative 0.02 to 0.02. The Luo estimator (blue dotted line) shows a logarithmic increase, plateauing near 0.015. Q E (green dashed), w Q E (red solid), and M D E (black dash-dot) remain near zero.

Top-right panel: Relative Bias in S D. The y-axis ranges from negative 0.050 to 0.050. Shi (blue dotted) increases from negative 0.025 toward 0.025. Q E (green dashed) decreases from 0.050 toward 0.025. w Q E (red solid) and M D E (black dash-dot) converge toward zero from negative values.

Bottom-left panel: R M S E in Mean. The y-axis ranges from 1 to 5. Luo (blue dotted) shows a steep linear increase from 1 to 5. Q E, w Q E, and M D E remain flat near 1.

Bottom-right panel: R M S E in S D. The y-axis ranges from 0 to 10. Q E (green dashed) shows a steep, jagged increase reaching 10. Shi (blue dotted) shows a moderate linear increase to 4. w Q E and M D E remain low, plateauing near 2.

Navigate back to Figure 2.

### Figure 3 Long description

A four-panel line graph display. All panels share an x-axis representing sample size n, ranging from 0 to 1000.

Top-Left Panel: Relative Bias in Mean. The y-axis ranges from negative 0.02 to 0.02. All four estimators (Luo, Q E, w Q E, M D E) converge rapidly toward zero bias as n increases, showing minor fluctuations below n equals 250.

Top-Right Panel: Relative Bias in S D. The y-axis ranges from negative 0.05 to 0.10. The Q E estimator (green dashed line) starts high at 0.10 and asymptotically decreases but remains significantly higher than others. Shi (blue dotted), w Q E (red solid), and M D E (black dash-dot) converge near zero by n equals 250.

Bottom-Left Panel: R M S E in Mean. The y-axis ranges from 0 to 9. The Q E estimator shows a linear increase in R M S E as n increases, reaching approximately 10. Luo, w Q E, and M D E remain flat and near zero across all values of n.

Bottom-Right Panel: R M S E in S D. The y-axis ranges from 0 to 16. The Q E estimator shows a steep linear increase, reaching 16 at n equals 1000. Shi, w Q E, and M D E show a very slight, near-horizontal increase, remaining below 4.

Navigate back to Figure 3.

### Figure 4 Long description

A four-panel grid of line graphs. All graphs share an x-axis labeled n, ranging from 0 to 1000.

Top-left panel: Relative Bias in Mean. The y-axis ranges from -0.10 to 0.05. The Q E estimator (green dashed line) starts at 0.04 and asymptotes toward 0.01. Shi (blue dots), w Q E (red solid), and M D E (black dash-dot) all converge quickly toward 0.

Top-right panel: Relative Bias in S D. The y-axis ranges from -0.1 to 0.2. Q E starts high at 0.2 and decreases to 0.08. Shi, w Q E, and M D E remain stable near 0.

Bottom-left panel: R M S T in Mean. The y-axis ranges from 1.0 to 3.0. Q E shows a steep linear increase from 1.5 to 3.0. w Q E and M D E show a moderate linear increase from 1.2 to 1.7. Shi remains the lowest and flattest, near 1.2.

Bottom-right panel: R M S E in S D. The y-axis ranges from 0 to 15. Q E shows a sharp linear increase from 5 to 18. Shi, w Q E, and M D E show very slight linear increases, remaining below 3.

Legend at the bottom of each panel: Shi (blue dotted line), Q E (green dashed line), w Q E (red solid line), M D E (black dash-dot line).

Navigate back to Figure 4.

### Figure 5 Long description

A four-panel set of line graphs. All graphs share an x-axis labeled n, ranging from 0 to 1000.

Top-left panel: Relative Bias in Mean. The y-axis ranges from -0.10 to 0.10. Luo (blue dotted line) shows a negative asymptotic curve starting at -0.01 and leveling near -0.07. Q E (green dashed line) stays slightly above 0. w Q E (red solid line) and M D E (black dash-dot line) converge near 0.

Top-right panel: Relative Bias in S D. The y-axis ranges from -0.1 to 0.2. Q E (green dashed line) starts high at 0.18 and decays toward 0.04. Shi (blue dotted line) remains constant near -0.05. w Q E and M D E converge just below 0.

Bottom-left panel: R M S E in Mean. The y-axis is labeled R M S T from 0 to 15. Luo (blue dotted line) shows a steep linear increase from 0 to 14. Q E, w Q E, and M D E remain flat near 0.

Bottom-right panel: R M S E in S D. The y-axis is labeled R M S E from 0 to 10.0. Q E (green dashed line) shows a steady linear increase from 4 to 10. Shi (blue dotted line), w Q E (red solid line), and M D E (black dash-dot line) all show a gradual linear increase from 1 to 3.5.

Navigate back to Figure 5.

### Figure 6 Long description

A four-panel multi-plot showing statistical estimator performance. All panels use n on the x-axis ranging from 0 to 1000.

* Top-left panel: Relative Bias in Mean. The y-axis ranges from -0.10 to 0.05. Luo (blue dots) shows a logarithmic curve starting at -0.01 and descending toward -0.07. Q E (green dashed), w Q E (red solid), and M D E (black dash-dot) all converge toward 0 as n increases.

* Top-right panel: Relative Bias in S D. The y-axis ranges from -0.1 to 0.2. Q E starts high at 0.15 and drops below 0. Shi (blue dots) remains constant near -0.03. w Q E and M D E converge quickly to 0.

* Bottom-left panel: R M S E in Mean. The y-axis ranges from 0 to 10. Luo shows a steep linear increase from 1 to 13. Q E, w Q E, and M D E remain flat and overlapping near 1.

* Bottom-right panel: R M S E in S D. The y-axis ranges from 0 to 12. Q E shows a significant linear increase from 3 to 11. Shi, w Q E, and M D E show much slower linear increases, remaining between 1 and 3.

Legend for Mean panels: Luo (blue dots), Q E (green dashed), w Q E (red solid), M D E (black dash-dot). Legend for S D panels: Shi (blue dots), Q E (green dashed), w Q E (red solid), M D E (black dash-dot).

Navigate back to Figure 6.

### Figure 7 Long description

The figure consists of four panels. All panels share an x-axis labeled n, ranging from 0 to 1000.

* Top-left panel: Relative Bias in Mean. The y-axis ranges from negative 0.2 to 0.1. Luo (blue dotted line) shows a logarithmic decrease from negative 0.02 to negative 0.18. Q E (green dashed), w Q E (red solid), and M D E (black dash-dot) all start near 0.1 and converge asymptotically toward 0 as n increases.

* Top-right panel: Relative Bias in S D. The y-axis ranges from negative 0.2 to 0.2. Shi (blue dotted) remains relatively flat near negative 0.1. Q E (green dashed) starts high at 0.2 and levels off near 0.05. w Q E (red solid) and M D E (black dash-dot) converge quickly to 0.

* Bottom-left panel: R M S E in Mean. The y-axis ranges from 0 to 30. Luo (blue dotted) shows a sharp linear increase from 0 to 30. Q E, w Q E, and M D E remain flat and near 0 across all values of n.

* Bottom-right panel: R M S E in S D. The y-axis ranges from 0 to 10. Q E (green dashed) starts very high, drops to 4, then gradually increases. Shi (blue dotted) shows a steady linear increase from 1 to 9. w Q E (red solid) and M D E (black dash-dot) remain stable and low near 2.

Navigate back to Figure 7.

### Table 2 Long description

The table is structured with a header row defining three conversion methods: mu sub 1, mu sub 2, and mu sub 3. Each method contains three sub-columns: Pooled estimate with 95 percent C I, tau, and I-squared.

There are five scenarios listed in the first column:

1. True sample mean/S D: Pooled estimate 6.53 (5.97, 7.09), tau 2.13, I-squared 97.4 percent across all three methods.

2. Luo/Shi (Wan): For mu sub 1, pooled estimate 5.76 (5.15, 6.37), tau 2.33, I-squared 98.5 percent. For mu sub 2, pooled estimate 5.68 (5.06, 6.29), tau 2.35, I-squared 97.8 percent. For mu sub 3, pooled estimate 5.97 (5.36, 6.58), tau 2.32, I-squared 98.1 percent.

3. Q E: For mu sub 1, pooled estimate 7.65 (7.08, 8.23), tau 2.15, I-squared 97.5 percent. For mu sub 2, pooled estimate 6.67 (5.99, 7.35), tau 2.58, I-squared 98.0 percent. For mu sub 3, pooled estimate 6.59 (6.03, 7.15), tau 2.13, I-squared 97.5 percent.

4. w Q E: For mu sub 1, pooled estimate 6.50 (5.93, 7.07), tau 2.18, I-squared 97.9 percent. For mu sub 2, pooled estimate 6.66 (5.97, 7.34), tau 2.60, I-squared 97.9 percent. For mu sub 3, pooled estimate 6.65 (6.11, 7.20), tau 2.07, I-squared 97.1 percent.

5. M D E: For mu sub 1, pooled estimate 6.51 (5.93, 7.09), tau 2.21, I-squared 98.0 percent. For mu sub 2, pooled estimate 6.60 (5.90, 7.30), tau 2.68, I-squared 98.1 percent. For mu sub 3, pooled estimate 6.71 (6.16, 7.25), tau 2.08, I-squared 97.8 percent.

Navigate back to Table 2.
