# Supplementary material for: Minimum distance estimation of mean and standard deviation from reported quantiles
Source: Res Synth Methods. 2026 Apr 15;17(5):958–75. doi: 10.1017/rsm.2026.10090 (PMC13732367; doi:10.1017/rsm.2026.10090)
Supplement: Supplementary file 1 [file S1759287926100908sup001.docx]

**Minimum Distance Estimation of Mean and Standard Deviation from Reported Quantiles**

**Supporting Materials**

Xiaoyu Tang^1^, Tiejun Tong^2^, Xin Zhang^3^, Haitao Chu^4,5^

^1^Academy of Pharmacy, Xi’an Jiaotong-Liverpool University, Suzhou, Jiangsu, China

^2^Department of Mathematics, Hong Kong Baptist University, Hong Kong

^3^Data Sciences and Analytics, Pfizer Inc., Shanghai, China

^4^Data Sciences and Analytics, Pfizer Inc., New York, NY, USA

^5^Division of Biostatistics and Health Data Science, University of Minnesota, Minneapolis, MN, USA

Correspondence: Haitao Chu, University of Minnesota, Minneapolis, MN 55455, USA; Email: chux0051@umn.edu

Text S1. Asymtotpic behaviors for weights of minimum and maximum.

We analyzed the asymptotic behavior of extreme quantile weights as $n\to\infty$. We define that the minimum has percentile $p_{a}=c/n$ and the maximum has percentile $p_{b}=1-c/n$ , where n is the sample size, and $c$ is a constant. In our methods, we adopted $c=0.625$.

**1. Normal Distribution:** $N(\mu,\sigma^{2})$

For the normal distribution, the probability density function (PDF) is $f(x)=(\sigma\sqrt{2\pi})^{-1}e^{-(x-\mu)^{2}/(2\sigma^{2})}$ and the quantile function is $F^{-1}(p)=\mu+\sigma\cdot\Phi^{-1}(p)$, where $\Phi$ denotes the standard normal cumulative distribution function.

**Minimum** : As $n\to\infty$, the standard normal quantile satisfies $\Phi^{-1}(c/n)\sim-\sqrt{2lnn-2lnc}$. Substituting into the quantile function yields:

$$x_{p_{a}}\sim\mu-\sigma\sqrt{2lnn-2lnc}$$

The density at $x_{p_{a}}$ evaluation gives:

$$f(x_{p_{a}})=\frac{1}{\sigma\sqrt{2\pi}}e^{-(lnn-lnc)}=\frac{1}{\sigma\sqrt{2\pi}}\cdot\frac{c}{n}$$

Therefore, $f(x_{p_{a}})=O(1/n)$, and the asymptotic weight satisfies:

$$\frac{[f(x_{p_{a}})]^{2}}{p_{a}(1-p_{a})}=O\left( \frac{1}{n} \right)$$

**Maximum:** By symmetry of the normal distribution, identical asymptotic behavior holds:

$$\frac{[f(x_{p_{b}})]^{2}}{p_{b}(1-p_{b})}=O\left( \frac{1}{n} \right)$$

**2. Log-Normal Distribution:** $LN(\mu,\sigma)$

For the log-normal distribution, the PDF is $f(x)=\frac{1}{x\sigma\sqrt{2\pi}}e^{-\frac{(lnx-\mu)^{2}}{2\sigma^{2}}}$ and quantile function is $F^{-1}(p)=exp(\mu+\sigma\cdot\Phi^{-1}(p))$.

**Minimum :** The quantile satisfies:

$$x_{p_{a}}\sim exp\left( \mu-\sigma\sqrt{2lnn-2lnc} \right)$$

The density at $x_{p_{a}}$ is:

$$f(x_{p_{a}})\sim\frac{1}{\sigma\sqrt{2\pi}}\cdot\frac{c}{n}\cdot exp\left( -\mu+\sigma\sqrt{2lnn-2lnc} \right)=O\left( \frac{1}{n}exp(\sigma\sqrt{2lnn}) \right)$$

The asymptotic weight satisfies:

$$\frac{[f(x_{p_{a}})]^{2}}{p_{a}(1-p_{a})}=\frac{\left[ O\left( \frac{1}{n}\exp\left( \sigma\sqrt{2\ln n} \right) \right) \right]^{2}}{c/n}=O\left( \frac{1}{n}exp(2\sigma\sqrt{2lnn}) \right)$$

We can express

$$exp(\sigma\sqrt{2lnn})=n^{\sigma\sqrt{2/lnn}}$$

Then

$$\frac{[f(x_{p_{a}})]^{2}}{p_{a}(1-p_{a})}=O\left( n^{-1+2\sigma\sqrt{2/lnn}} \right)$$

**Maximum:** The quantile behaves as:

$$x_{p_{b}}\sim exp\left( \mu+\sigma\sqrt{2lnn-2lnc} \right)$$

The density satisfies:

$$f(x_{p_{b}})\sim\frac{1}{\sigma\sqrt{2\pi}}\cdot\frac{c}{n}\cdot exp\left( -\sigma\sqrt{2lnn} \right)=O\left( \frac{1}{n}exp(-\sigma\sqrt{2lnn}) \right)$$

Thus, the asymptotic weight is:

$$\frac{[f(x_{p_{b}})]^{2}}{p_{b}(1-p_{b})}=O\left( \frac{1}{n}exp(-2\sigma\sqrt{2\ln n} \right)=O\left( n^{-1-2\sigma\sqrt{2/lnn}} \right)$$

**3. Weibull Distribution:** $Weibull(k,\lambda)$

For the Weibull distribution, the PDF is $f(x)=\frac{k}{\lambda}(x/\lambda)^{k-1}e^{-(x/\lambda)^{k}}$ and quantile function is $x_{p}=\lambda(-ln(1-p))^{1/k}$.

**Minimum:** Using the asymptotic equivalence $-ln(1-p)\sim p$ for $p\to0$:

$$x_{p_{a}}\sim\lambda(c/n)^{1/k}$$

The density is:

$$f\left( x_{p_{a}} \right)=\frac{k}{\lambda}\left( \frac{x_{p_{a}}}{\lambda} \right)^{k-1}e^{-(x_{p_{a}}/\lambda)^{k}}\sim\frac{k}{\lambda}(c/n)^{(k-1)/k}=O(n^{-(k-1)/k})$$

Therefore, the weight satisfies:

$$\frac{[f(x_{p_{a}})]^{2}}{p_{a}(1-p_{a})}=O(n^{(2-k)/k})$$

**Maximum:** The quantile is:

$$x_{p_{b}}=\lambda(-ln(c/n))^{1/k}$$

Noting that $(x_{p_{b}}/\lambda)^{k}=-ln(c/n)$ and $\left( \frac{x_{p_{b}}}{\lambda} \right)^{k-1}=(lnn-lnc)^{(k-1)/k}$, the density is:

$$f\left( x_{p_{b}} \right)=\frac{k}{\lambda}\left( \frac{x_{p_{b}}}{\lambda} \right)^{k-1}e^{-(x_{p_{b}}/\lambda)^{k}}\sim\frac{k}{\lambda}(lnn)^{(k-1)/k}\cdot\frac{c}{n}=O\left( n^{-1}(lnn)^{(k-1)/k} \right)$$

Thus, the asymptotic weight is:

$$\frac{[f(x_{p_{b}})]^{2}}{p_{b}(1-p_{b})}=O\left( n^{-1}(lnn)^{2(k-1)/k} \right)$$

**4. Gamma Distribution:** $Gamma(\alpha,\beta)$

For the Gamma distribution, the PDF $f(x)=\frac{\beta^{\alpha}}{\Gamma(\alpha)}x^{\alpha-1}e^{-\beta x}$ and CDF $F(x)=\frac{\gamma(\alpha,\beta x)}{\Gamma(\alpha)}$, where $\gamma(\alpha,z)$ is the lower incomplete Gamma function.

**Minimum:** Using the small-argument expansion of the incomplete gamma function:

$$F(x)\sim\frac{\beta^{\alpha}}{\Gamma(\alpha)}\cdot\frac{x^{\alpha}}{\alpha}=\frac{c}{n}$$

Solving the equation yields $x_{p_{a}}\sim\left( \frac{\alpha\Gamma(\alpha)c}{\beta^{\alpha}n} \right)^{1/\alpha}$. The density satisfies:

$$f\left( x_{p_{a}} \right)=\frac{\beta^{\alpha}}{\Gamma\left( \alpha\right)}x_{p_{a}}^{\alpha-1}e^{-\beta x_{p_{a}}}\sim\frac{\beta^{\alpha}}{\Gamma\left( \alpha\right)}\left( \frac{\alpha\Gamma\left( \alpha\right)c}{\beta^{\alpha}n} \right)^{\frac{\alpha-1}{\alpha}}=O(n^{-(\alpha-1)/\alpha})$$

Therefore, the weight behaves as:

$$\frac{[f(x_{p_{a}})]^{2}}{p_{a}(1-p_{a})}=O(n^{(2-\alpha)/\alpha})$$

**Maximum:** Using the first-order approximation of the expansion for the upper incomplete gamma function for large arguments:

$$1-F(x)\sim\frac{(\beta x)^{\alpha-1}e^{-\beta x}}{\Gamma(\alpha)}=\frac{c}{n}$$

The solution of this equation, let $z=\beta x_{p_{b}}$,

$$z-\left( \alpha-1 \right)\ln z=lnn-ln(c\Gamma(\alpha))$$

We expect $z$to be of order $\ln\left( n \right)$, so we substitute $z=lnn+\delta$, then:

$$\left( \ln n+\delta\right)-\left( \alpha-1 \right)\ln\left( \ln n+\delta\right)=lnn-ln(c\Gamma(\alpha))$$

The quantile will behave as:

$$x_{p_{b}}\sim\frac{1}{\beta}\left[ lnn+(\alpha-1)ln(lnn)-ln(c\Gamma(\alpha)) \right]$$

As

$$e^{-\beta x_{p_{b}}}\sim e^{-lnn-(\alpha-1)ln(lnn)+ln(c\Gamma(\alpha))}=\frac{c\Gamma(\alpha)}{n(lnn)^{\alpha-1}}$$

The density is:

$$f(x_{p_{b}})\sim\frac{\beta^{\alpha}}{\Gamma\left( \alpha\right)}\cdot\left( \frac{\ln n}{\beta} \right)^{\alpha-1}\cdot\frac{c\Gamma\left( \alpha\right)}{n(lnn)^{\alpha-1}}=\frac{\beta c}{n}=O\left( \frac{1}{n} \right)$$

Thus, the weight is:

$$\frac{[f(x_{p_{b}})]^{2}}{p_{b}(1-p_{b})}=O\left( \frac{1}{n} \right)$$

**5. Beta Distribution:** $Beta(\alpha,\beta)$

For the Beta distribution, the PDF is $f(x)=\frac{1}{B(\alpha,\beta)}x^{\alpha-1}(1-x)^{\beta-1}$ and the CDF is F$(x)=\frac{1}{B\left( \alpha,\beta\right)}\int_{0}^{x} t^{\alpha-1}\left( 1-t \right)^{\beta-1}dt$

**Minimum:** Using the small-argument approximation of the incomplete Beta function:

$$F(x)\sim\frac{1}{B(\alpha,\beta)}\cdot\frac{x^{\alpha}}{\alpha}=\frac{c}{n}$$

Solving the equation yields $x_{p_{a}}\sim\left( \frac{\alpha B(\alpha,\beta)c}{n} \right)^{1/\alpha}$. The density satisfies:

$$f(x_{p_{a}})\sim\frac{1}{B(\alpha,\beta)}x_{p_{a}}^{\alpha-1}=\frac{1}{B(\alpha,\beta)}\left( \frac{\alpha B(\alpha,\beta)c}{n} \right)^{(\alpha-1)/\alpha}=O(n^{-(\alpha-1)/\alpha})$$

Therefore, the weight behaves as:

$$\frac{[f(x_{p_{a}})]^{2}}{p_{a}(1-p_{a})}=O(n^{(2-\alpha)/\alpha})$$

**Maximum:** Let $y=1-x$. Then $y\sim Beta(\beta,\alpha)$ and:

$$F_{Y}(y)\sim\frac{1}{B(\alpha,\beta)}\cdot\frac{y^{\beta}}{\beta}=\frac{c}{n}$$

Solving the equation gives $y\sim\left( \frac{\beta B(\alpha,\beta)c}{n} \right)^{1/\beta}$, and thus:

$$x_{p_{b}}=1-y\sim1-\left( \frac{\beta B(\alpha,\beta)c}{n} \right)^{1/\beta}$$

The density is:

$$f(x_{p_{b}})\frac{1}{B(\alpha,\beta)}x_{p_{b}}^{\alpha-1}y^{\beta-1}\sim\frac{1}{B(\alpha,\beta)}y^{\beta-1}=O(n^{-(\beta-1)/\beta})$$

Thus, the weight is:

$$\frac{[f(x_{p_{b}})]^{2}}{p_{b}(1-p_{b})}=O(n^{(2-\beta)/\beta})$$

Figure S1. Performance of the QE method with different percentiles for minimum under $Beta(min=0,max=8,\alpha=4, \beta=2)$.

Figure S2. Performance of the QE method with different percentiles for minimum under $Normal\left( 5,3 \right).$

Figure S3. Performance of the QE method with different percentiles for minimum under $Lognormal(1.5, 0.5)$.

Figure S4. Performance of the QE method with different percentiles for minimum under $Gamma\left( 2.5, 0.5 \right).$

Figure S5. Performance of the QE method with different percentiles for minimum under $Weibull\left( 1.5, 5 \right).$

Figure S6. Performance of the QE method with different percentiles for minimum under $Exponential(0.2).$

Figure S7. Performance of estimators under $Beta(min=0,max=8,\alpha=4, \beta=2)$ in $S_{1}$. Least squares method was used for distribution selection for wQE and MDE.

Figure S8. Performance of estimators under $Normal(5,3)$ in $S_{1}$. Least squares method was used for distribution selection for wQE and MDE.

Figure S9. Performance of estimators under $Lognormal(1.5, 0.5)$ in $S_{1}$. Least squares method was used for distribution selection for wQE and MDE.

Figure S10. Performance of estimators under $Gamma(2.5, 0.5)$ in $S_{1}$. Least squares method was used for distribution selection for wQE and MDE.

Figure S11. Performance of estimators under $Weibull(1.5, 5)$ in $S_{1}$. Least squares method was used for distribution selection for wQE and MDE.

Figure S12. Performance of estimators under $Exponential(0.2)$ in $S_{1}$. Least squares method was used for distribution selection for wQE and MDE.

Figure S13. Performance of estimators under $Beta(min=0,max=8,\alpha=4, \beta=2)$ in $S_{2}$. Least squares method was used for distribution selection for wQE and MDE.

Figure S14. Performance of estimators under $Normal(5,3)$ in $S_{2}$. Least squares method was used for distribution selection for wQE and MDE.

Figure S15. Performance of estimators under $Lognormal(1.5, 0.5)$ in $S_{2}$. Least squares method was used for distribution selection for wQE and MDE.

Figure S16. Performance of estimators under $Gamma(2.5, 0.5)$ in $S_{2}$. Least squares method was used for distribution selection for wQE and MDE.

Figure S17. Performance of estimators under $Weibull(1.5, 5)$ in $S_{2}$. Least squares method was used for distribution selection for wQE and MDE.

Figure S18. Performance of estimators under $Exponential(0.2)$ in $S_{2}$. Least squares method was used for distribution selection for wQE and MDE.

Figure S19. Proportion of correct selection of distributions using least squares in $S_{3}$.

Figure S20. Proportion of correct selection of distributions using least squares in $S_{2}$.

Figure S21. Proportion of correct selection of distributions using least squares in $S_{1}$.

Figure S22. Performance of estimators under $Beta(min=0,max=8,\alpha=4, \beta=2)$ in $S_{3}$. Least absolute deviations method was used for distribution selection for wQE and MDE.

Figure S23. Performance of estimators under $Normal(5,3)$ in $S_{3}$. Least absolute deviations method was used for distribution selection for wQE and MDE.

Figure S24. Performance of estimators under $Lognormal(1.5, 0.5)$ in $S_{3}$. Least absolute deviations method was used for distribution selection for wQE and MDE.

Figure S25. Performance of estimators under $Gamma(2.5, 0.5)$ in $S_{3}$. Least absolute deviations method was used for distribution selection for wQE and MDE.

Figure S26. Performance of estimators under $Weibull(1.5, 5)$ in $S_{3}$. Least absolute deviations method was used for distribution selection for wQE and MDE.

Figure S27. Performance of estimators under $Exponential(0.2)$ in $S_{3}$. Least absolute deviations method was used for distribution selection for wQE and MDE.

Figure S28. Performance of estimators under $Beta(min=0,max=8,\alpha=4, \beta=2)$ in $S_{2}$. Least absolute deviations method was used for distribution selection for wQE and MDE.

Figure S29. Performance of estimators under $Normal(5,3)$ in $S_{2}$. Least absolute deviations method was used for distribution selection for wQE and MDE.

Figure S30. Performance of estimators under $Lognormal(1.5, 0.5)$ in $S_{2}$. Least absolute deviations method was used for distribution selection for wQE and MDE.

Figure S31. Performance of estimators under $Gamma(2.5, 0.5)$ in $S_{2}$. Least absolute deviations method was used for distribution selection for wQE and MDE.

Figure S32. Performance of estimators under $Weibull(1.5, 5)$ in $S_{2}$. Least absolute deviations method was used for distribution selection for wQE and MDE.

Figure S33. Performance of estimators under $Exponential(0.2)$ in $S_{2}$. Least absolute deviations method was used for distribution selection for wQE and MDE.

Figure S34. Performance of estimators under $Beta(min=0,max=8,\alpha=4, \beta=2)$ in $S_{1}$. Least absolute deviations method was used for distribution selection for wQE and MDE.

Figure S35. Performance of estimators under $Normal(5,3)$ in $S_{1}$. Least absolute deviations method was used for distribution selection for wQE and MDE.

Figure S36. Performance of estimators under $Lognormal(1.5, 0.5)$ in $S_{1}$. Least absolute deviations method was used for distribution selection for wQE and MDE.

Figure S37. Performance of estimators under $Gamma(2.5, 0.5)$ in $S_{1}$. Least absolute deviations method was used for distribution selection for wQE and MDE.

Figure S38. Performance of estimators under $Weibull(1.5, 5)$ in $S_{1}$. Least absolute deviations method was used for distribution selection for wQE and MDE.

Figure S39. Performance of estimators under $Exponential(0.2)$ in $S_{1}$. Least absolute deviations method was used for distribution selection for wQE and MDE.

Figure S40. Proportion of correct selection of distributions using least absolute deviations in $S_{3}$.

Figure S41. Proportion of correct selection of distributions using least absolute deviations in $S_{2}$.

Figure S42. Proportion of correct selection of distributions using least absolute deviations in $S_{1}$.

Figure S43. Performance of estimators under $Weibull(0.8, 5)$ in $S_{3}$. Least absolute deviations method was used for distribution selection for wQE and MDE.

Figure S44. Performance of estimators under $Gamma(0.8, 5)$ in $S_{3}$. Least absolute deviations method was used for distribution selection for wQE and MDE.

Figure S45. Performance of estimators under $Beta(0.8, 4)$ in $S_{3}$. Least absolute deviations method was used for distribution selection for wQE and MDE.

Figure S46. Performance of estimators under $Beta(4, 0.8)$ in $S_{3}$. Least absolute deviations method was used for distribution selection for wQE and MDE.

Table S1. Mean and SD of distributions in simulations.

| Distribution | Mean | SD |
| --- | --- | --- |
| Beta(min=0, max=8,4,2) | 5.33 | 1.43 |
| Gamma (2.5, 0.5) | 5.00 | 3.16 |
| Lognormal (1.5, 0.5) | 5.08 | 2.71 |
| Normal (5,3) | 5.00 | 3.00 |
| Weibull (1.5, 5) | 4.51 | 3.06 |
| Exponential (0.2) | 5.00 | 5.00 |

Table S2. Data for PHQ-9 scores in real data example.

| Study | N | Min | Q1 | Median | Q3 | Max | Mean | 95%CI Lower Bound | 95% CI Upper bound |
| --- | --- | --- | --- | --- | --- | --- | --- | --- | --- |
| Persoons et al. 2001 | 173 | 0 | 2 | 5 | 9 | 27 | 6.4 | 5.55 | 7.24 |
| Henkel et al. 2004 | 430 | 0 | 3 | 5 | 10 | 25 | 7.01 | 6.46 | 7.56 |
| Grafe et al. 2004 | 494 | 0 | 3 | 7 | 12 | 27 | 8.12 | 7.59 | 8.65 |
| Fann et al. 2005 | 135 | 0 | 0 | 4 | 8.5 | 24 | 5.59 | 4.45 | 6.53 |
| Picardi et al. 2005 | 138 | 0 | 2 | 5 | 10 | 25 | 6.76 | 5.78 | 7.74 |
| Azah et al. 2005 | 180 | 0 | 3 | 5 | 8 | 21 | 5.91 | 5.33 | 6.49 |
| Hahn et al. 2006 | 211 | 0 | 5.5 | 9 | 14 | 26 | 9.61 | 8.82 | 10.39 |
| Eack et al. 2006 | 48 | 1 | 4 | 9 | 16.25 | 24 | 10.06 | 8.03 | 12.1 |
| Muramatsu et al. 2007 | 116 | 0 | 3 | 7 | 13 | 27 | 8.92 | 7.6 | 10.24 |
| Stafford et al. 2007 | 193 | 0 | 1 | 3 | 7 | 27 | 4.84 | 4.04 | 5.64 |
| Hides et al. 2007 | 103 | 0 | 6 | 13 | 18.5 | 27 | 12.62 | 11.18 | 14.06 |
| Patel et al. 2008 | 299 | 0 | 1 | 4 | 7 | 27 | 5.07 | 4.52 | 5.62 |
| Thombs et al. 2008 | 1006 | 0 | 1 | 3 | 8 | 25 | 5.19 | 4.85 | 5.53 |
| Lotrakul et al. 2008 | 278 | 0 | 3 | 6 | 9 | 24 | 6.51 | 6 | 7.01 |
| Lamers et al. 2008 | 104 | 0 | 3 | 5 | 12 | 27 | 7.15 | 5.96 | 8.34 |
| Wittkampf et al. 2009 | 260 | 0 | 1 | 4 | 9 | 27 | 5.72 | 4.99 | 6.44 |
| Osorio et al. 2009 | 177 | 0 | 1 | 5 | 14 | 24 | 7.79 | 6.69 | 8.88 |
| Gjerdingen et al. 2009 | 419 | 0 | 1 | 3 | 6 | 27 | 4.38 | 3.93 | 4.83 |
| Richardson et al. 2010 | 377 | 0 | 3 | 7 | 11 | 27 | 7.77 | 7.19 | 8.34 |
| van Steenbergen-Weijenburg et al. 2010 | 196 | 0 | 2 | 7.5 | 12 | 27 | 7.94 | 7.03 | 8.85 |
| Arroll et al. 2010 | 2528 | 0 | 1 | 3 | 6 | 27 | 4.29 | 4.1 | 4.48 |
| Ayalon et al. 2010 | 151 | 0 | 0 | 2 | 5 | 24 | 5.91 | 5.33 | 6.49 |
| Delgadillo et al. 2011 | 103 | 0 | 10 | 13 | 17.5 | 27 | 13.1 | 11.96 | 14.24 |
| Hyphantis et al. 2011 | 213 | 0 | 2 | 5 | 9.5 | 23 | 6.33 | 5.65 | 7.01 |
| Hobfoll et al. 2011 | 144 | 0 | 1 | 4 | 10 | 26 | 5.94 | 4.97 | 6.9 |
| Khamseh et al. 2011 | 184 | 0 | 6 | 11 | 19 | 27 | 12.25 | 11.15 | 13.35 |
| Liu et al. 2011 | 1532 | 0 | 0 | 2 | 5 | 25 | 3.38 | 3.18 | 3.58 |
| Pence et al. 2012 | 398 | 0 | 0 | 1 | 4 | 19 | 2.58 | 2.22 | 2.94 |
| Osorio et al. 2012 | 86 | 0 | 4.25 | 9 | 15.75 | 27 | 10.41 | 8.92 | 11.9 |
| Mohd Sidik et al. 2012 | 146 | 0 | 2 | 3 | 7 | 21 | 4.73 | 4.03 | 5.43 |
| Bombardier et al. 2012 | 160 | 0 | 2 | 5 | 10 | 27 | 6.66 | 5.84 | 7.49 |
| Sidebottom et al. 2012 | 246 | 0 | 2 | 5 | 9 | 26 | 6.4 | 5.68 | 7.11 |
| Turner et al. 2012 | 72 | 0 | 2.75 | 6 | 10 | 26 | 7.24 | 5.85 | 8.62 |
| Williams et al. 2012 | 235 | 0 | 2 | 5 | 8 | 21 | 5.85 | 5.23 | 6.47 |
| de Man-van Ginkel et al. 2012 | 164 | 0 | 3 | 6 | 10 | 23 | 7.04 | 6.21 | 7.86 |
| Simning et al. 2012 | 190 | 0 | 2 | 4 | 7.75 | 21 | 5.09 | 4.48 | 5.7 |
| Kwan et al. 2012 | 113 | 0 | 2 | 4 | 8 | 27 | 5.5 | 4.53 | 6.46 |
| Sung et al. 2013 | 399 | 0 | 1 | 3 | 6 | 27 | 4.34 | 3.92 | 4.76 |
| Inagaki et al. 2013 | 104 | 0 | 0 | 2 | 3.19 | 22 | 2.79 | 2.07 | 3.5 |
| Razykov et al. 2013 | 345 | 0 | 3 | 6 | 10 | 26 | 2.79 | 2.07 | 3.5 |
| Rooney et al. 2013 | 126 | 0 | 3 | 5 | 9 | 25 | 6.82 | 6.25 | 7.38 |
| Vohringer et al. 2013 | 190 | 0 | 5 | 8 | 14 | 27 | 6.71 | 5.74 | 7.67 |
| Zhang et al. 2013 | 68 | 0 | 2 | 5 | 10 | 26 | 9.86 | 8.97 | 10.76 |
| Twist et al. 2013 | 360 | 0 | 0 | 2 | 7 | 27 | 4.47 | 3.9 | 5.03 |
| Chagas et al. 2013 | 84 | 0 | 4 | 7.5 | 12 | 23 | 8.43 | 7.15 | 9.71 |
| Akena et al. 2013 | 91 | 0 | 2 | 6 | 9 | 23 | 6.33 | 5.23 | 7.43 |
| Santos et al. 2013 | 196 | 0 | 1 | 4 | 8 | 21 | 5.64 | 4.91 | 6.37 |
| McGuire et al. 2013 | 100 | 0 | 1 | 4 | 8.5 | 23 | 5.61 | 4.46 | 6.76 |
| Fischer et al. 2014 | 194 | 0 | 1 | 4 | 8 | 27 | 5.48 | 4.7 | 6.27 |
| Gelaye et al. 2014 | 923 | 0 | 2 | 5 | 10 | 27 | 6.55 | 6.21 | 6.9 |
| Beraldi et al. 2014 | 116 | 0 | 3 | 6 | 8 | 16 | 6 | 5.31 | 6.69 |
| Cholera et al. 2014 | 397 | 0 | 2 | 5 | 9 | 22 | 6.12 | 5.65 | 6.59 |
| Fiest et al. 2014 | 169 | 0 | 1 | 4 | 9 | 26 | 5.68 | 4.84 | 6.52 |
| Hyphantis et al. 2014 | 349 | 0 | 2 | 5 | 10 | 27 | 6.76 | 6.13 | 7.39 |
| Kiely et al. 2014 | 822 | 0 | 1 | 3 | 6 | 27 | 4.14 | 3.84 | 4.44 |
| Lambert et al. 2015 | 147 | 0 | 2 | 6 | 10 | 24 | 6.84 | 5.93 | 7.76 |
| Amoozegar et al. 2017 | 203 | 0 | 3 | 7 | 12 | 27 | 8.06 | 7.22 | 8.91 |
| Turner et al. Unpublished | 51 | 0 | 0.5 | 3 | 5 | 24 | 4.12 | 2.71 | 5.52 |

Source of dataset: McGrath S, Zhao X, Steele R, et al. Estimating the sample mean and standard deviation from commonly reported quantiles in meta-analysis. *Stat Methods Med Res* 2020; 29: 2520-2537. 20200130. DOI: 10.1177/0962280219889080.

Table S3. Distribution selection for different methods in real data example under $S_{1}$.

| Methods | Weibull | Beta | Normal | Lognormal | Gamma |
| --- | --- | --- | --- | --- | --- |
| QE | 5.2% | 93.1% | 0.0% | 0.0% | 1.7% |
| wQE | 44.8% | 46.6% | 8.6% | 0.0% | 0.0% |
| MDE | 46.6% | 44.8% | 8.6% | 0.0% | 0.0% |

Table S4. Distribution selection for different methods in real data example under $S_{2}$.

| Methods | Weibull | Beta | Normal | Lognormal | Gamma |
| --- | --- | --- | --- | --- | --- |
| QE | 6.9% | 39.7% | 24.1% | 15.5% | 13.8% |
| wQE | 6.9% | 32.8% | 27.6% | 13.8% | 19.0% |
| MDE | 8.6% | 29.3% | 31.0% | 12.1% | 19.0% |

Table S5. Distribution selection for different methods in real data example under $S_{3}$.

| Methods | Weibull | Beta | Normal | Lognormal | Gamma |
| --- | --- | --- | --- | --- | --- |
| QE | 43.1% | 41.4% | 1.7% | 0.0% | 13.8% |
| wQE | 10.3% | 82.8% | 1.7% | 0.0% | 5.2% |
| MDE | 8.6% | 84.5% | 3.5% | 0.0% | 3.5% |
